# Comprehensive evaluation of prophylactic HPV vaccines: a systematic review and meta-analysis of efficacy, safety, and immunogenicity in males and females

**DOI:** 10.3389/fimmu.2025.1747082

**Published:** 2026-01-13

**Authors:** Zhiliang Wang, Hui Zhang, Yuhang Zhu, Yuanli Jiang, Danhe Yang, Xiaofeng Zou, Yi Fang

**Affiliations:** 1 Department of Obstetrics and Gynecology, Affiliated Hospital of Zunyi Medical University, Zunyi, China; 2 Clinical Trial Institution, Peking University People’s Hospital, Beijing, China; 3 Department of Pharmacy Administration and Clinical Pharmacy, School of Pharmaceutical Sciences, Peking University, Beijing, China; 4 Early Clinical Research Ward, Affiliated Hospital of Zunyi Medical University, Zunyi, China

**Keywords:** cervical intraepithelial neoplasia, HPV vaccines, human papillomavirus, immunogenicity, meta-analysis, vaccine safety

## Abstract

**Background:**

Human papillomavirus (HPV) vaccination is a cornerstone of global strategies to prevent HPV-associated malignancies; however, uncertainties persist regarding its long-term efficacy, immunogenicity, and safety across populations, vaccine formulations, and dosing schedules. We conducted a comprehensive systematic review and meta-analysis to evaluate the clinical and immunological effectiveness of prophylactic HPV vaccines.

**Methods:**

This study followed PRISMA guidelines and was registered in PROSPERO (CRD420251050526). Randomized controlled trials (RCTs) were identified through comprehensive searches of six major databases. Risk of bias was assessed using the RoB 2 tool. Meta-analyses were performed using random- or fixed-effects models as appropriate. Subgroup and meta-regression analyses explored the effects of age, sex, vaccine type, dosing regimen, and HIV status. Certainty of evidence was appraised using the GRADE framework.

**Results:**

A total of 145 RCTs were included. HPV vaccination significantly reduced cervical intraepithelial neoplasia grade 1 (RR = 0.15, 95% CI: 0.09–0.24), grade 2 (0.20, 0.13–0.30), and grade 3 (0.48, 0.23–0.98). Persistent HPV16/18 infections were reduced by 84% (0.16, 0.12–0.21), and incident infections by 75% (0.25, 0.19–0.34). Immunogenicity analyses demonstrated robust antibody responses, with fold increases of 3.09 for HPV16, 3.10 for HPV18, 3.48 for HPV6, and 3.37 for HPV11. Tissue-resident CD4^+^ T cells were significantly reduced (SMD: −1.27, 95% CI: −2.24 to −0.31), while CD8^+^ T cells showed no significant change. Vaccination was not associated with an increased risk of serious adverse events (0.90, 0.82–0.99) or adverse pregnancy outcomes. Although, injection-site adverse events were modestly increased (1.26, 1.07–1.48).

**Conclusion:**

Prophylactic HPV vaccination provides strong protection against HPV infections and precancerous lesions and induces robust humoral immunity with a favorable safety profile. The nonavalent vaccine, particularly when administered in a three-dose (0/1/6) regimen, offers the most comprehensive protection. These findings support the expansion of gender-neutral vaccination programs to maximize population-level cancer prevention.

**Systematic review registration:**

https://www.crd.york.ac.uk/prospero/, identifier CRD420251050526.

## 1 Introduction

Human papillomavirus (HPV) is a major global health concern and the leading cause of cervical, anal, oropharyngeal, and other malignancies (1). The advent of prophylactic HPV vaccines has revolutionized cancer prevention, significantly reducing persistent HPV infections and high-grade precancerous lesions (2). Despite this success, several fundamental questions remain unanswered regarding the long-term efficacy, comparative immunogenicity, and safety of different vaccine formulations (3). While prior systematic reviews and meta-analyses have provided valuable insights, they suffer from notable limitations that restrict their generalizability and clinical applicability.

Many studies have focused predominantly on female cohorts, neglecting the vaccine’s efficacy in males and other high-risk populations, leading to an incomplete understanding of its broader impact (4, 5). Furthermore, variations in vaccine response across different age groups and among immunocompromised individuals, particularly those living with HIV, remain insufficiently explored (6, 7). While HPV vaccines have demonstrated strong safety profiles, inconsistencies in adverse event reporting across different vaccine types have led to ambiguity in risk assessments, with some reviews failing to systematically compare vaccine-related adverse effects (8). Additionally, significant heterogeneity in study designs, follow-up durations, and endpoints used to measure vaccine efficacy has made it difficult to establish conclusive evidence regarding long-term protection and the prevention of high-grade precancerous lesions (9, 10). Some analyses have also been limited in scope, focusing on specific vaccine types while neglecting a direct comparative evaluation of the bivalent, quadrivalent, and nonavalent vaccines (11). Moreover, the role of HPV vaccination in reducing recurrent disease following surgical treatment for HPV-related lesions remains an area of debate, with conflicting findings across studies (8, 10).

Considering the global prioritization of HPV immunization programs and the urgent need for evidence-based policy recommendations, a rigorous and comprehensive systematic review and meta-analysis is imperative. To the best of our knowledge, no prior study has simultaneously evaluated the efficacy, immunogenicity, and safety of all major prophylactic HPV vaccines across both sexes, multiple vaccine formulations, and various high-risk populations. By synthesizing the highest-quality evidence available, this study aims to provide a definitive comparative evaluation that will guide vaccination policies, optimize public health strategies, and contribute to the long-term reduction of HPV-related malignancies.

## 2 Methods

This research has been officially recorded in the International Prospective Register of Systematic Reviews (PROSPERO) with the identifier CRD420251050526 and adheres strictly to the Preferred Reporting Items for Systematic Reviews and Meta-Analyses (PRISMA) 2020 guidelines for systematic reviews and meta-analyses (12).

### 2.1 Search strategy

A comprehensive literature search was conducted across Web of Science, PubMed, Embase, Scopus, Cochrane Library, and ClinicalTrials.gov, covering studies from January 1, 2010, to January 31, 2025. The temporal restriction from 2010 onward was applied to prioritize contemporary randomized evidence reflecting current vaccine formulations, standardized outcome reporting, and populations relevant to present vaccination policies. The search utilized Medical Subject Headings (MeSH) related to papillomavirus infections, HPV vaccines (such as Gardasil and Cervarix), immunization, and randomized controlled trials. Additionally, bibliographic references of the selected studies were examined to identify further relevant articles.

### 2.2 Eligibility principles

The selection criteria were established following the PICO (Population, Intervention, Comparison, Outcome) framework. The study included both males and females across different age groups with documented HPV serostatus (either seropositive or seronegative) and HIV infection status. The intervention was limited to prophylactic HPV vaccination using one of the following formulations: bivalent (HPV16 and HPV18), quadrivalent (HPV6, HPV11, HPV16, and HPV18), or nonavalent (HPV6, HPV11, HPV16, HPV18, HPV31, HPV33, HPV45, HPV52, and HPV58) vaccines. Vaccination regimens included single and two-dose schedules (administered at varying intervals, such as months 0 and 2, 0 and 6, or 0 and 12) and three-dose schedules, either at standard intervals (e.g., months 0, 1, and 6 or 0, 2, and 6) or extended intervals (e.g., months 0, 2, and 12). The comparison group received either a placebo (e.g., saline solution or an adjuvant-containing formulation) or an active control vaccine (e.g., hepatitis B virus [HBV] or hepatitis A virus [HAV] vaccines), administered at the same dosage and schedule as the intervention group. The full list of main outcomes is provided in **Supplementary Table S1**.

Studies were excluded if they did not specifically evaluate HPV vaccination, involved inappropriate interventions in either the experimental or control groups, included therapeutic HPV vaccines or prophylactic vaccines administered with a therapeutic intent, were unpublished, did not adhere to a randomized controlled trial (RCT) design, or failed to report at least one predefined main outcome measure.

### 2.3 Data extraction

Titles and abstracts were independently screened by two reviewers Zhiliang Wang and Hui Zhang), with discrepancies resolved by a third investigator (Danhe Yang). Extracted data included the first author’s surname, year of publication, patient characteristics, sample size, and intervention details (dose, type, duration). For studies with multiple experimental arms, data from each arm were extracted separately. When multiple publications reported outcomes from the same trial cohort, data were extracted from the most recent and comprehensive report to avoid duplicate inclusion and to capture longer-term follow-up.

### 2.4 Bias and quality control

Two independent evaluators (Zhiliang Wang and Hui Zhang) conducted a review of study reliability and bias risk, with a third reviewer (Yuhang Zhu) resolving any discrepancies. The Cochrane RoB 2 tool was employed to assess the quality of randomized controlled trials (RCTs). To examine publication bias, funnel plots were analyzed visually, and Egger’s regression test was performed when the meta-analysis included at least 10 studies. If any asymmetry was detected, the trim-and-fill approach was implemented to correct for potential publication bias. Moreover, a leave-one-out sensitivity analysis was conducted to determine the effect of individual studies on the overall findings.

### 2.5 Subgroup and meta-regression analysis

Subgroup analyses and meta-regression evaluations were performed following predefined selection criteria for meta-analyses that included a minimum of 10 studies.

### 2.6 Certainty assessment

The strength of the evidence was assessed using the GRADE (Grading of Recommendations, Assessment, Development, and Evaluation) framework, which classifies certainty into four levels: high, moderate, low, and very low. Because all included studies were randomized controlled trials, evidence for each outcome was initially rated as high certainty and subsequently downgraded at the outcome level based on prespecified criteria. Downgrading was applied for risk of bias (informed by RoB 2 assessments), inconsistency (substantial heterogeneity, I² > 50%), imprecision (wide confidence intervals or limited sample sizes), and publication bias (funnel plot asymmetry or significant Egger’s test results when applicable). Indirectness was evaluated by examining the applicability of populations, interventions, comparators, and outcomes to the review question. All certainty judgments followed consistent decision rules to enhance transparency and reproducibility.

### 2.7 Statistical analysis

Data was analyzed statistically with RevMan 5.4 (Nordic Cochran Centre, Copenhagen, Denmark) and Stata 14 (College Station, TX, USA). This study calculated the pooled risk ratio (RR) for dichotomous data as well as the pooled standardized mean difference (SMD) for continuous data, accompanied by 95% confidence intervals (CIs). Fold changes in geometric mean titers (GMTs) and their corresponding 95% CIs were derived from log-transformed GMT ratios to improve precision and comparability across studies. Inter-study heterogeneity was assessed using I² statistics. A fixed-effects model was applied, whenever there was no significant heterogeneity (I² < 50% or P > 0.05); otherwise, a random-effects model was applied. Reference management and data organization were handled using EndNote 21. A p-value < 0.05 was deemed to be statistically meaningful.

## 3 Results

### 3.1 Study selection

**Figure 1:**
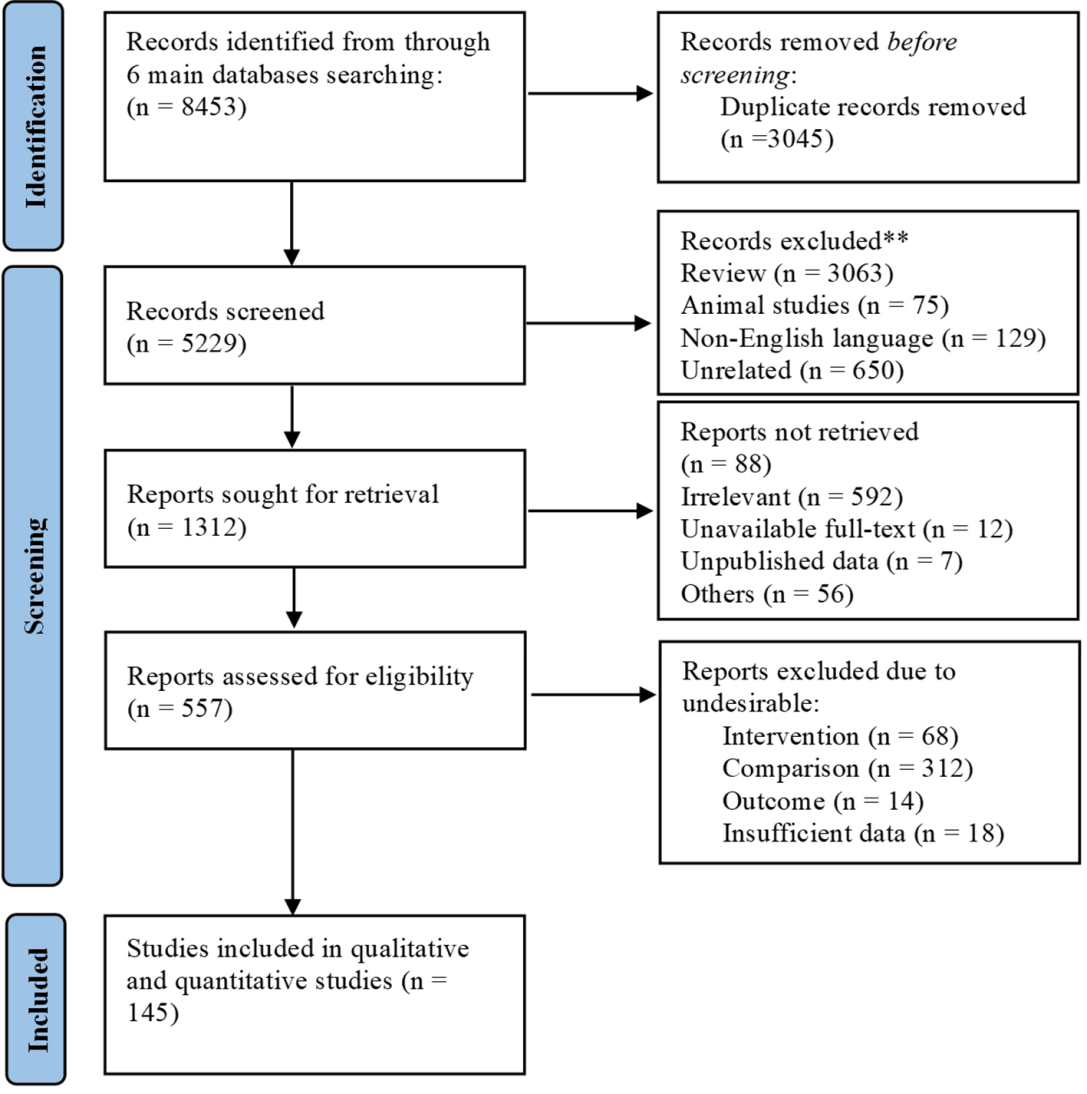
PRISMA flow diagram illustrating the study selection process.

Of 8,453 identified records, 3,045 duplicates were removed, leaving 5,229 for screening. After excluding 3,917 records due to review articles (3,063), animal studies (75), non-English language (129) and unrelated topics (650), 1,312 full-text articles were assessed. Among these, 88 reports were not retrieved due to irrelevance (592), unavailable full-text (12), unpublished data (7), or other reasons (56). After assessing 557 articles for eligibility, 412 were excluded due to undesirable intervention (68), comparison (312), outcome (14), or insufficient data (18). Ultimately, 145 (13–58) studies were included (**Figure 1**).

### 3.2 Study characteristics

**Figure 2:**
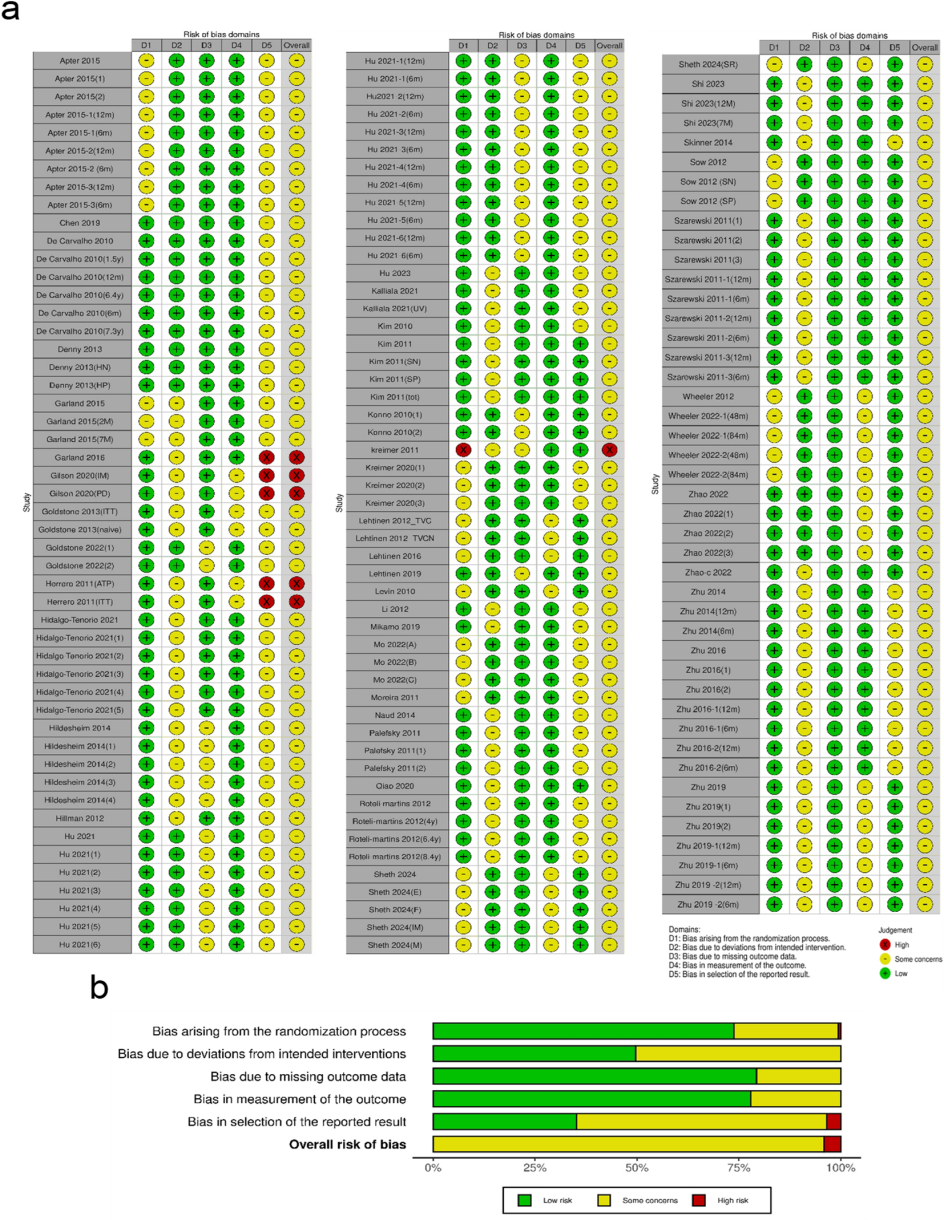
**(a)** Summary and **(b)** graphical representation of the risk of bias assessment.

This meta-analysis included 145 RCTs published between 2010 and 2024, evaluating the efficacy, safety, and immunogenicity of bivalent, quadrivalent, and nonavalent prophylactic HPV vaccines. Studies assessed single-dose, two-dose, and three-dose regimens across diverse populations. The majority were conducted in China (50 studies) and multinational settings (40 studies). Treatment duration ranged from 1 to 16 months, with follow-up periods extending from 6 to 136 months. While most trials focused on female participants, some included male cohorts. Immunogenicity assessments included both humoral responses, measured by HPV-specific antibody titers, and cellular immunity, evaluating CD4^+^ and CD8^+^ T-cell responses. Safety outcomes encompassed systemic and injection-site adverse events (**Supplementary Table S2**). Risk of bias assessment indicated that 95% of studies had “Some Concerns,” with six classified as “High Risk,” primarily due to selective outcome reporting (**Figure 2**).

### 3.3 Cervical intraepithelial neoplasia grade 1

**Figure 3:**
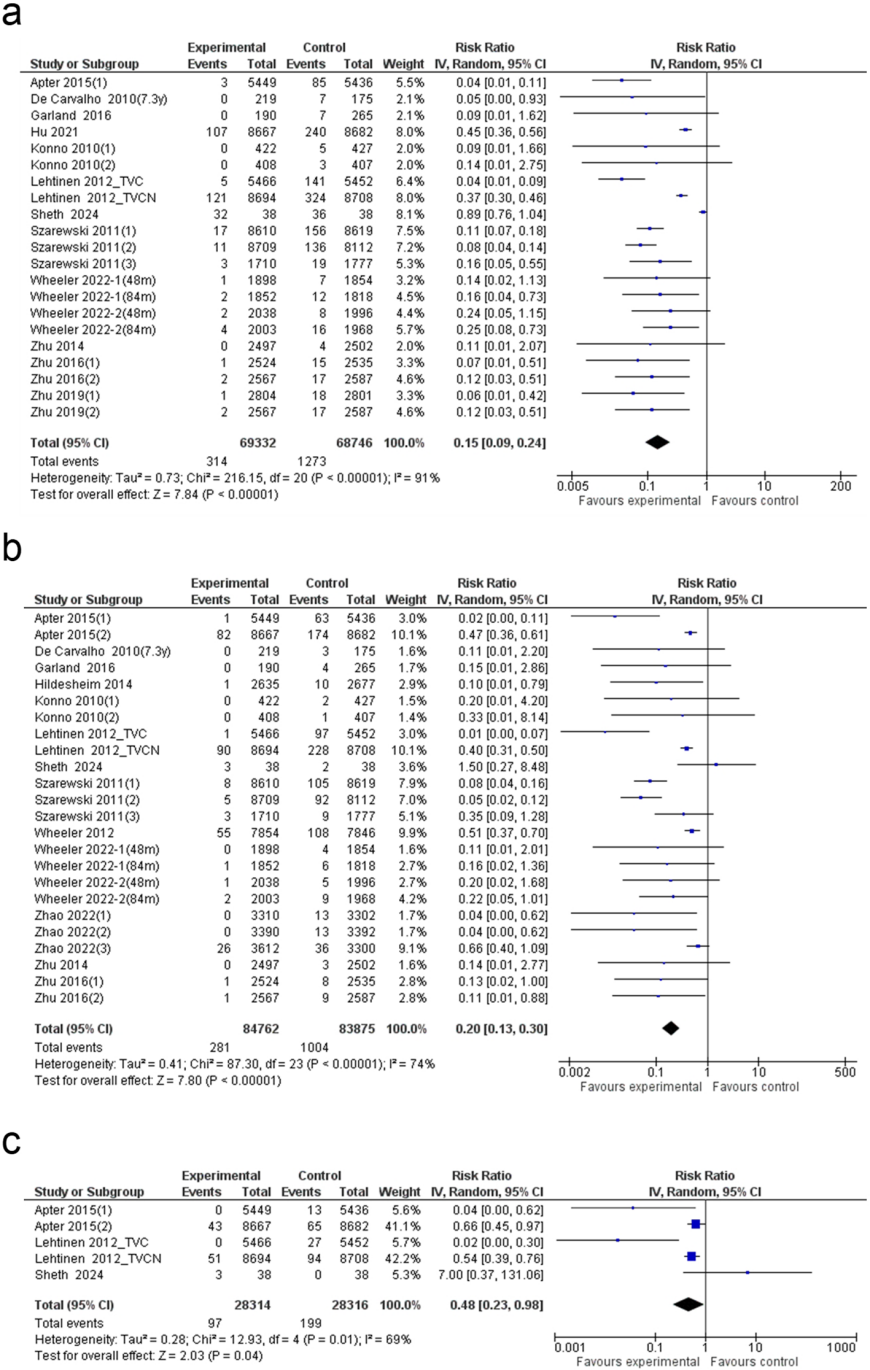
Forest plot of the **(a)** CIN I, **(b)** CIN II and **(c)** CIN III. CIN: cervical intraepithelial neoplasia grade.

The meta-analysis of 21 studies demonstrated a notable reduction in CIN I risk with the HPV vaccination compared to the control (RR: 0.15; 95% CI: 0.09 to 0.24; p < 0.00001), though heterogeneity was substantial (I² = 91%) (**Figure 3A**). Removal of high–risk-of-bias studies yielded effect estimates and heterogeneity comparable to the primary analysis (RR ≈ 0.15; I² ≈ 91%), suggesting that study quality did not account for the substantial heterogeneity observed. The funnel plot indicated a mild asymmetry, suggesting potential publication bias (**Supplementary Figure S1A**). Egger’s test confirmed bias (p = 0.040), reinforcing the asymmetry detected in the funnel plot (**Supplementary Table 3**). Post trim-and-fill adjustment, the pooled estimate remained unchanged (**Supplementary Table S4**), indicating robust results despite bias concerns. The most substantial CIN I reduction was observed in individuals ≤ 20 years old (88% decrease). The three-dose (0/1/6) regimen showed the highest efficacy (87% reduction), while among vaccine types, the quadrivalent vaccine provided the greatest protection (95% reduction) (**Supplementary Figure S2**). Meta-regression revealed no statistically significant associations across age, dose regimen, or vaccine type, suggesting that none independently accounted for variability in CIN I reduction (**Supplementary Table S5**).

### 3.4 CIN II

Meta-analysis of 24 studies showed a significant reduction in CIN II risk following HPV vaccination (80% reduction; 95% CI: 70–87%), with moderate heterogeneity (I² = 74%) (**Figure 3B**). The funnel plot appeared symmetric, suggesting no substantial publication bias (**Supplementary Figure S1B**), which was further supported by a non-significant Egger’s test (p = 0.057) (**Supplementary Table S3**). Subgroup analysis identified a significant effect of dose regimen, with the three-dose (0/1/6) schedule yielding the greatest risk reduction (**Supplementary Figure S3**). Among vaccine types, the bivalent vaccine demonstrated the highest efficacy in reducing CIN II associated with HPV 16/18. Meta-regression analysis revealed no statistically significant predictors, indicating that none of the examined factors independently accounted for variability in CIN II reduction (**Supplementary Table S5**).

### 3.5 CIN III

Analysis of five studies revealed a significant 52% reduction in CIN III risk following HPV vaccination (95% CI: 2–77%). Heterogeneity was moderate (I² = 69%), and the overall effect reached statistical significance (p = 0.04), confirming the protective impact of HPV vaccination against CIN III (**Figure 3C**).

### 3.6 Persistent infections of HPV 16/18

**Figure 4:**
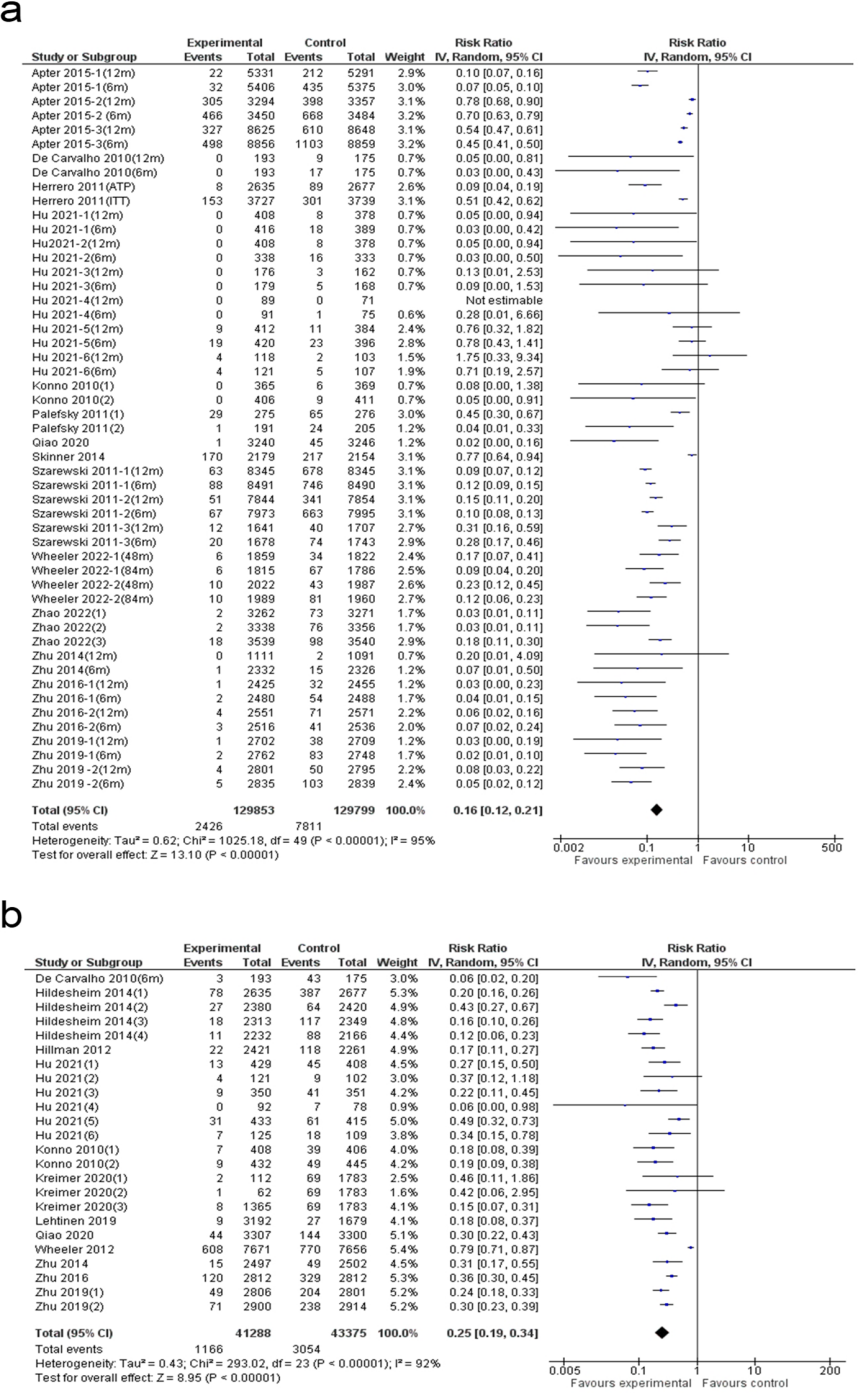
Forest plot of the **(a)** persistent infections and **(b)** incident infections.

A comprehensive meta-analysis of 51 studies identified a notable reduction in persistent HPV 16/18 infections following HPV vaccination (84% reduction; 95% CI: 79–88%), with high heterogeneity (I² = 95%) (**Figure 4A**). The funnel plot indicated asymmetry, suggesting potential publication bias (**Supplementary Figure S1C**), which was confirmed by Egger’s test (p = 0.008) (**Supplementary Table S3**). Trim-and-fill analysis did not adjust the pooled estimate, indicating that publication bias had minimal impact on the overall findings (**Supplementary Table S4**). Subgroup analysis showed no statistically significant differences across evaluated factors, and meta-regression identified no significant predictors, suggesting that none independently accounted for the observed variation in persistent HPV 16/18 infections (**Supplementary Table S5** and **Supplementary Figure S4**).

### 3.7 Incident infections of HPV 16/18

A detailed meta-analysis involving 24 studies identified a notable decline in incident HPV 16/18 infections following HPV vaccination (75% reduction; 95% CI: 66–81%), with substantial heterogeneity (I² = 92%) (**Figure 4B**). Funnel plot analysis indicated asymmetry, pointing to the potential presence of publication bias (**Supplementary Figure S1D**). This finding was further verified through Egger’s test, which yielded a statistically significant result (p = 0.001), indicating the presence of small-study effects (**Supplementary Table S3**). Trim-and-fill analysis did not adjust the pooled estimate, suggesting that while publication bias was detected, it had minimal impact on the overall findings (**Supplementary Table S4**). Subgroup analysis identified HIV status as a significant factor, with the HIV-negative group experiencing the greatest reduction in incident infections (94% reduction) (**Supplementary Figure S5**). No statistically significant differences were observed across other subgroup categories, and meta-regression revealed no significant predictors, indicating that none independently accounted for the variation in incident HPV 16/18 infections (**Supplementary Table S5**).

### 3.8 Risk of atypical squamous cells of undetermined significance

**Figure 5:**
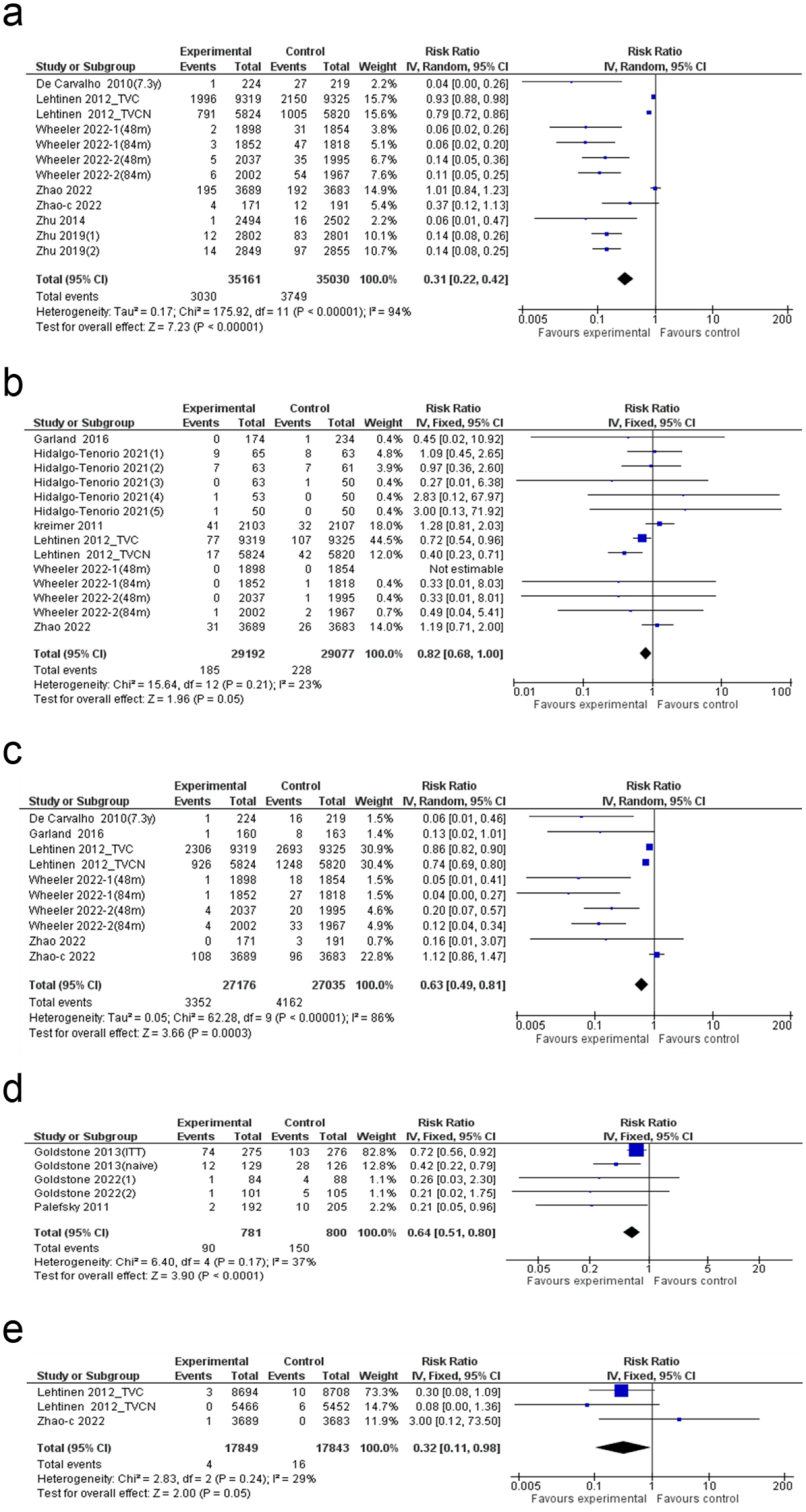
Forest plot of the **(a)** ASC-US, **(b)** HSIL, **(c)** LSIL, **(d)** AIN and **(e)** AIS. ASC-US: atypical squamous cells of undetermined significance; HSIL: high-grade squamous intraepithelial lesion; LSIL: low-grade squamous intraepithelial lesion; AIN: anal intraepithelial neoplasia; AIS: adenocarcinoma in situ.

Meta-analysis of 10 studies demonstrated a significant reduction in ASC-US risk following HPV vaccination (69% reduction; 95% CI: 58–81%), with high heterogeneity (I² = 94%) (**Figure 5A**). The funnel plot exhibited slight asymmetry, suggesting potential publication bias (**Supplementary Figure S1E**). Also, Egger’s test indicated the presence of bias (p = 0.004) (**Supplementary Table S3**). Trim-and-fill analysis did not alter the pooled estimate, suggesting that although publication bias was detected, its impact on the overall findings was minimal (**Supplementary Table S4**). Subgroup analysis revealed that the greatest reduction in ASC-US risk was observed in individuals aged 20–30 years (87% reduction), while among HIV status categories, the most pronounced effect was seen in the HIV-negative group (96% reduction) (**Supplementary Figure S6**). Meta-regression analysis found no significant associations, indicating that none of the examined variables independently influenced ASC-US risk reduction (**Supplementary Table S5**).

### 3.9 Risk of high-grade squamous intraepithelial lesion

Meta-analysis of 14 studies demonstrated a moderate reduction in HSIL risk following HPV vaccination (18% reduction; 95% CI: 0–32%), with low heterogeneity (I² = 23%) (**Figure 5B**). The overall effect was borderline significant (p = 0.05). The funnel plot appeared symmetric, suggesting no major publication bias (**Supplementary Figure S1F**). Egger’s test was not statistically significant (p = 0.573), further confirming the absence of small-study effects (**Supplementary Table S3**).

### 3.10 Low-grade squamous intraepithelial lesion risk analysis

A systematic review of 10 studies identified a notable decline in LSIL risk among participants in the experimental group compared to the control group (RR: 0.63; 95% CI: 0.49 to 0.81; p = 0.0003), with substantial heterogeneity (I² = 86%) (**Figure 5C**). The funnel plot suggested slight asymmetry, raising concerns about potential publication bias (**Supplementary Figure S1G**). However, Egger’s test (p = 0.178) found no statistically significant indication of publication bias (**Supplementary Table S3**). Subgroup analysis showed the highest LSIL risk reduction in the HIV-negative group (94%) and the bivalent vaccine group (47%) (**Supplementary Figure S7**). However, meta-regression analysis across these subgroup criteria showed no statistically significant associations (**Supplementary Table S5**).

### 3.11 Anal intraepithelial neoplasia risk analysis

A meta-analysis including six studies found a significant reduction in AIN risk among individuals who received the HPV vaccine, compared to those in the control group (RR: 0.64; 95% CI: 0.51 to 0.80; p < 0.0001), with moderate heterogeneity (I² = 37%) (**Figure 5D**).

### 3.12 Adenocarcinoma *in situ* risk analysis

The meta-analysis of three studies indicated a notable decrease in AIS risk among individuals receiving the HPV vaccine in comparison with those in the control group (RR: 0.32; 95% CI: 0.11 to 0.98; p = 0.05), with low heterogeneity (I² = 29%) (**Figure 5E**).

### 3.13 External genital lesions risk analysis

**Figure 6:**
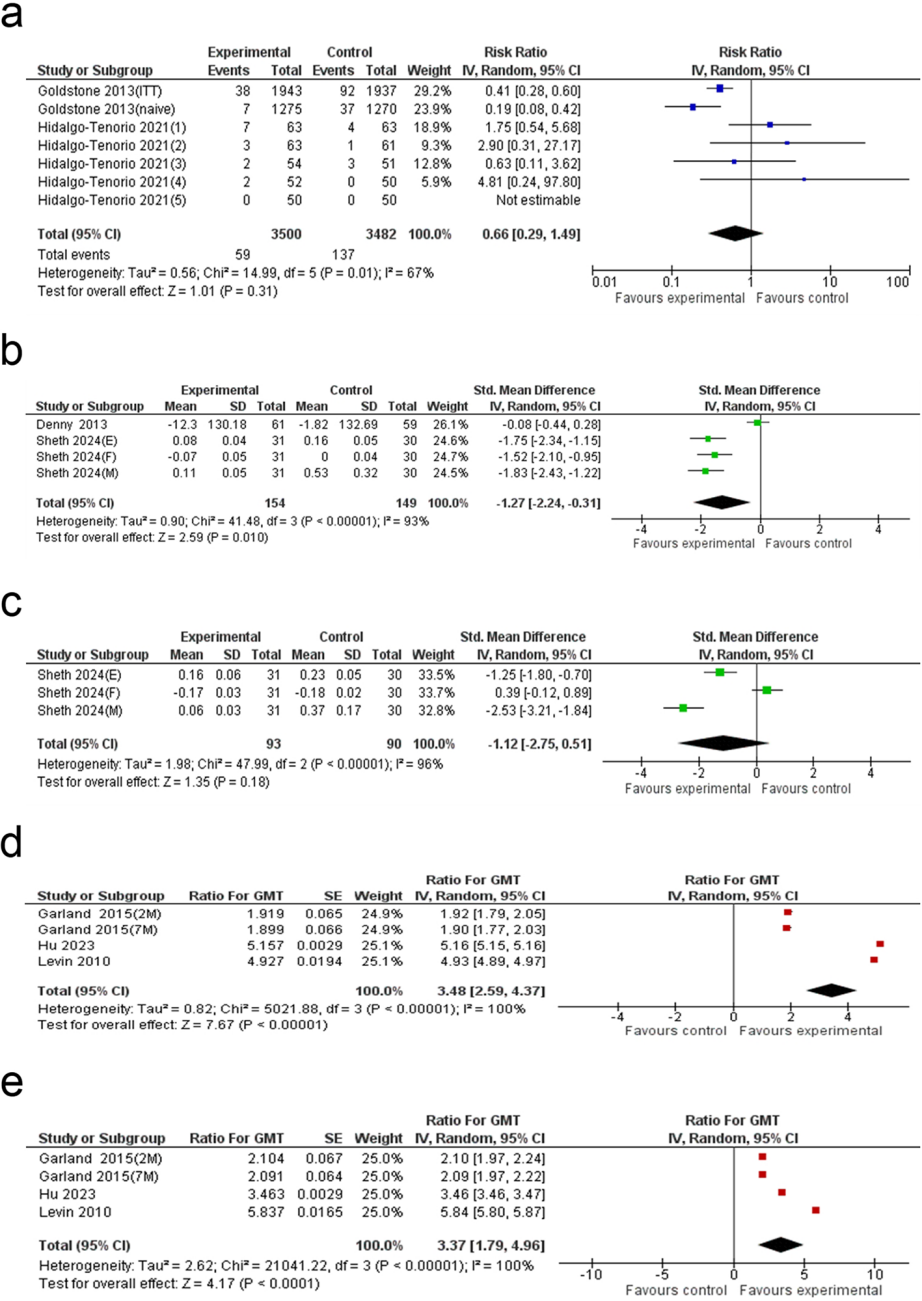
Forest plot of the **(a)** EGL, **(b)** TCD4+ cell count, **(c)** TCD8+ cell count, **(d)** GMT6 and **(e)** GMT11. EGL: external genital lesions; GMT6: geometric mean titer of anti HPV6 antibody; GMT11: geometric mean titer of anti HPV11 antibody.

The meta-analysis of seven studies showed no statistically significant difference in EGL risk between the HPV vaccine group and the control group (RR: 0.66; 95% CI: 0.29 to 1.49; p = 0.31), with moderate heterogeneity (I² = 67%) (**Figure 6A**).

### 3.14 Tissue CD4^+^ T cell count

A systematic review of four studies identified a significant decline in tissue-resident CD4^+^ T cell levels among participants who received the HPV vaccine compared to those in the control group (SMD: -1.27; 95% CI: -2.24 to -0.31; p = 0.010), with substantial heterogeneity (I² = 93%) (**Figure 6B**).

### 3.15 Tissue CD8^+^ T cell count

The meta-analysis of three studies showed no significant difference in tissue-resident CD8^+^ T cell levels between the HPV vaccine group and the control group (SMD: -1.12; 95% CI: -2.75 to 0.51; p = 0.18), with high heterogeneity (I² = 96%) (**Figure 6C**).

### 3.16 Anti-HPV6 antibody level

The meta-analysis of four studies demonstrated a 3.48-fold increase in anti-HPV6 antibody levels in the HPV vaccine group compared to the control (Ratio for GMT: 3.48; 95% CI: 2.59 to 4.37; p < 0.00001), with substantial heterogeneity (I² = 100%) (**Figure 6D**).

### 3.17 Anti-HPV11 antibody level

The meta-analysis of four studies showed a 3.37-fold increase in anti-HPV11 antibody levels in the HPV vaccine group compared to the control (Ratio for GMT: 3.37; 95% CI: 1.79 to 4.96; p < 0.0001), with substantial heterogeneity (I² = 100%) (**Figure 6E**).

### 3.18 Anti-HPV16 antibody levels

**Figure 7:**
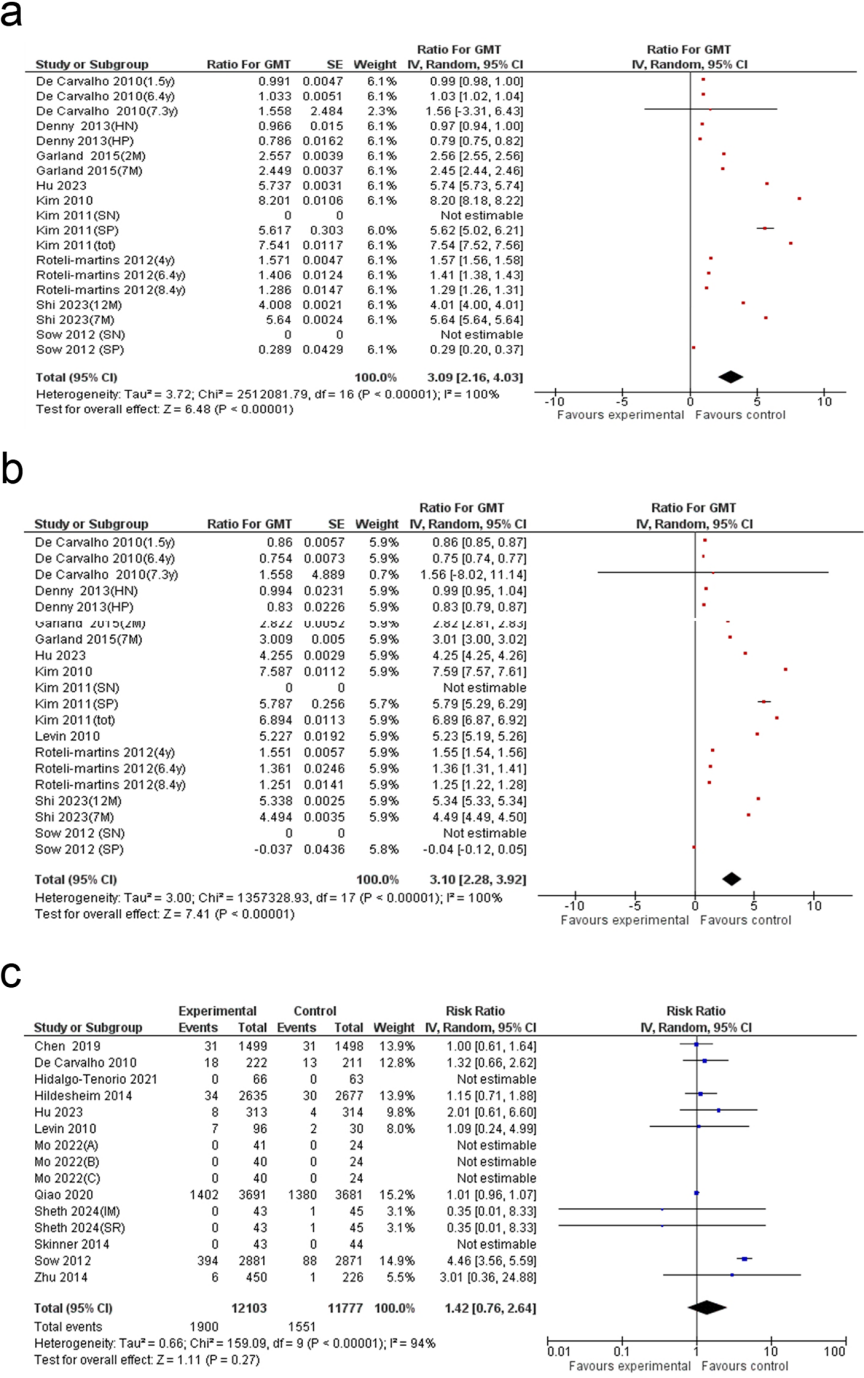
Forest plot of the **(a)** GMT16, **(b)** GMT18 and **(c)** grade 3 adverse events. GMT16: geometric mean titer of anti HPV16 antibody; GMT18: geometric mean titer of anti HPV18 antibody.

The meta-analysis of nineteen studies showed a 3.09-fold increase in anti-HPV16 antibody levels in the HPV vaccine group compared to the control (Ratio for GMT: 3.09; 95% CI: 2.16 to 4.03; p < 0.00001), with substantial heterogeneity (I² = 100%) (**Figure 7A**). The funnel plot did not reveal substantial asymmetry (**Supplementary Figure S1H**), and Egger’s test confirmed the absence of significant publication bias (p = 0.297) (**Supplementary Table S3**). Subgroup analysis showed the highest increase in anti-HPV16 antibody levels in the ≥30 years age group (5.13-fold, 95% CI: 3.95–6.31), followed by individuals aged ≤20 years (3.37-fold, 95% CI: 1.58–5.17) (**Supplementary Figure S8**). Antibody titers were higher in HIV-negative individuals (2.01-fold, 95% CI: 0.64–3.37) compared with HIV-positive individuals (0.88-fold, 95% CI: 0.70–1.05). Moreover, across included studies, higher fold changes were observed in sex-not reported (NR) studies (5.74-fold, 95% CI: 5.73–5.74), followed by female-only studies (2.92-fold, 95% CI: 1.95–3.88). Consistently, anti-HPV16 antibody levels increased across assay types, including enzyme-linked immunosorbent assay (ELISA) (2.65-fold, 95% CI: 1.33–3.97), chemiluminescent Immunoassay (CLIA) (2.50-fold, 95% CI: 2.40–2.61), and neutralization assays (5.13-fold, 95% CI: 3.95–6.31). Although, meta-regression based on these subgroup criteria did not yield significant results (**Supplementary Table S5**).

### 3.19 Anti-HPV18 antibody levels

The meta-analysis of 20 studies showed a 3.10-fold increase in anti-HPV18 antibody levels in the HPV vaccine group compared to the control (Ratio for GMT: 3.10; 95% CI: 2.28 to 3.92; p < 0.0001), with substantial heterogeneity (I² = 100%) (**Figure 7B**). Egger’s test and funnel plot suggested potential publication bias (p = 0.005) (**Supplementary Table S3** and **Supplementary Figure S1I**), while trim-and-fill analysis did not adjust the pooled estimate, indicating robustness of the results (**Supplementary Table S4**). Subgroup analysis indicated that the largest increase was observed in the ≥30 years age group (4.70-fold, 95% CI: 3.99–5.40). Across sex-based categories, studies enrolling both female and male participants demonstrated the greatest statistically significant increase (5.23-fold, 95% CI: 4.01–6.45), followed by studies with sex- NR cohort (5.06-fold, 95% CI: 3.91–6.20), whereas female-only studies showed a comparatively lower increase (2.85-fold, 95% CI: 1.92–3.78). Additionally, the subgroup with unspecified HIV status (NR) exhibited the greatest fold change in antibody titers (5.06-fold, 95% CI: 3.91–6.20). Although these increases were consistently observed across assay types, higher fold changes were detected in neutralization assays (4.70-fold, 95% CI: 3.95–5.40) than in CLIA and ELISA methods (**Supplementary Figure S9**). Meta-regression based on these subgroup criteria did not yield significant results (**Supplementary Table S5**).

### 3.20 Total Grade 3 adverse events

A meta-analysis encompassing 15 studies found no statistically significant variation in the incidence of Grade 3 adverse events between individuals in the HPV vaccine group and those in the control group (RR: 1.42; 95% CI: 0.76 to 2.64; p = 0.27). The analysis reported high heterogeneity (I² = 94%) (**Figure 7C**). Funnel plot analysis showed no major asymmetry (**Supplementary Figure S1J**), while Egger’s test confirmed that significant publication bias was not present (p = 0.565) (**Supplementary Table S3**). Additionally, subgroup analyses, including stratification by follow-up duration, and meta-regression did not yield statistically meaningful outcomes (**Supplementary Table S5** and **Supplementary Figure S10**).

### 3.21 Total serious adverse events

**Figure 8:**
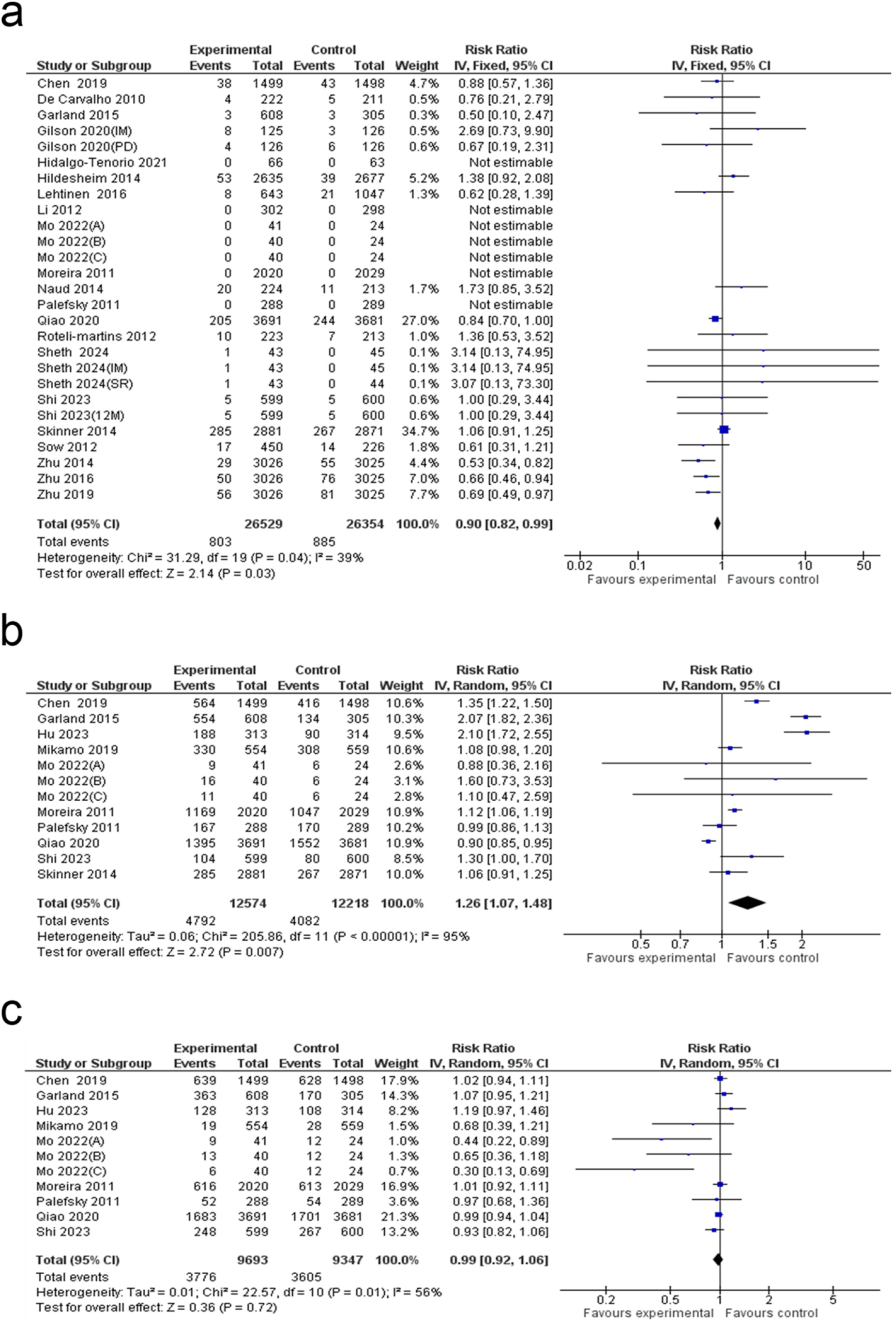
Forest plot of the **(a)** serious adverse events, **(b)** injection-site adverse events and **(c)** systemic adverse events.

A meta-analysis involving 27 studies found a 10% decrease in the occurrence of serious adverse events among individuals in the HPV vaccine group compared to those in the control group (RR: 0.90; 95% CI: 0.82 to 0.99; p = 0.03). The analysis reported moderate heterogeneity (I² = 39%) (**Figure 8A**). The funnel plot exhibited a predominantly symmetrical distribution (**Supplementary Figure S1K**), and Egger’s test (p = 0.291) confirmed the absence of significant publication bias (**Supplementary Table S3**).

### 3.22 Injection-site adverse events

A meta-analysis of 12 studies indicated a 26% increase in the rate of injection-site adverse events in individuals who received the HPV vaccine compared to those in the control group (RR: 1.26; 95% CI: 1.07 to 1.48; p = 0.007). The analysis found high heterogeneity (I² = 95%) (**Figure 8B**). The funnel plot revealed a mostly symmetrical pattern (**Supplementary Figure S11**), while Egger’s test (p = 0.164) confirmed no significant evidence of publication bias (**Supplementary Table S3**). Subgroup analysis showed that sex-stratified effects were significant (P < 0.00001), with males exhibiting the lowest and only statistically significant risk estimate. Further, among vaccine formulations, the bivalent vaccine was associated with the lowest risk estimate (RR: 1.05) (**Supplementary Figure S11**). Although no significant variation in local adverse events was observed across follow-up durations. Meta-regression analysis further identified a significant association between vaccine type and injection-site adverse events (β = 0.43; 95% CI: 0.17 to 0.70; p = 0.005) (**Supplementary Table S5**).

### 3.23 Systemic adverse events

The meta-analysis of 11 studies found no significant difference in the incidence of systemic adverse events between the HPV vaccine group and the control (RR: 0.99; 95% CI: 0.92 to 1.06; p = 0.72), with moderate heterogeneity (I² = 56%) (**Figure 8C**). The funnel plot appeared symmetric, suggesting minimal risk of publication bias (**Supplementary Figure S1M**). Egger’s test further confirmed the absence of significant publication bias (p = 0.126) (**Supplementary Table S3**). Subgroup analysis by sex revealed a significant difference, with studies enrolling both female and male participants showing the lowest risk of systemic adverse events (RR: 0.48; 95% CI: 0.31–0.73), whereas no significant associations were observed in the remaining sex-defined subgroups (**Supplementary Figure S12**). Although the risk of systemic adverse events did not show a meaningful change with longer follow-up. Meta-regression analysis did not identify any significant associations (**Supplementary Table S5**).

### 3.24 Pregnancy outcomes

**Figure 9:**
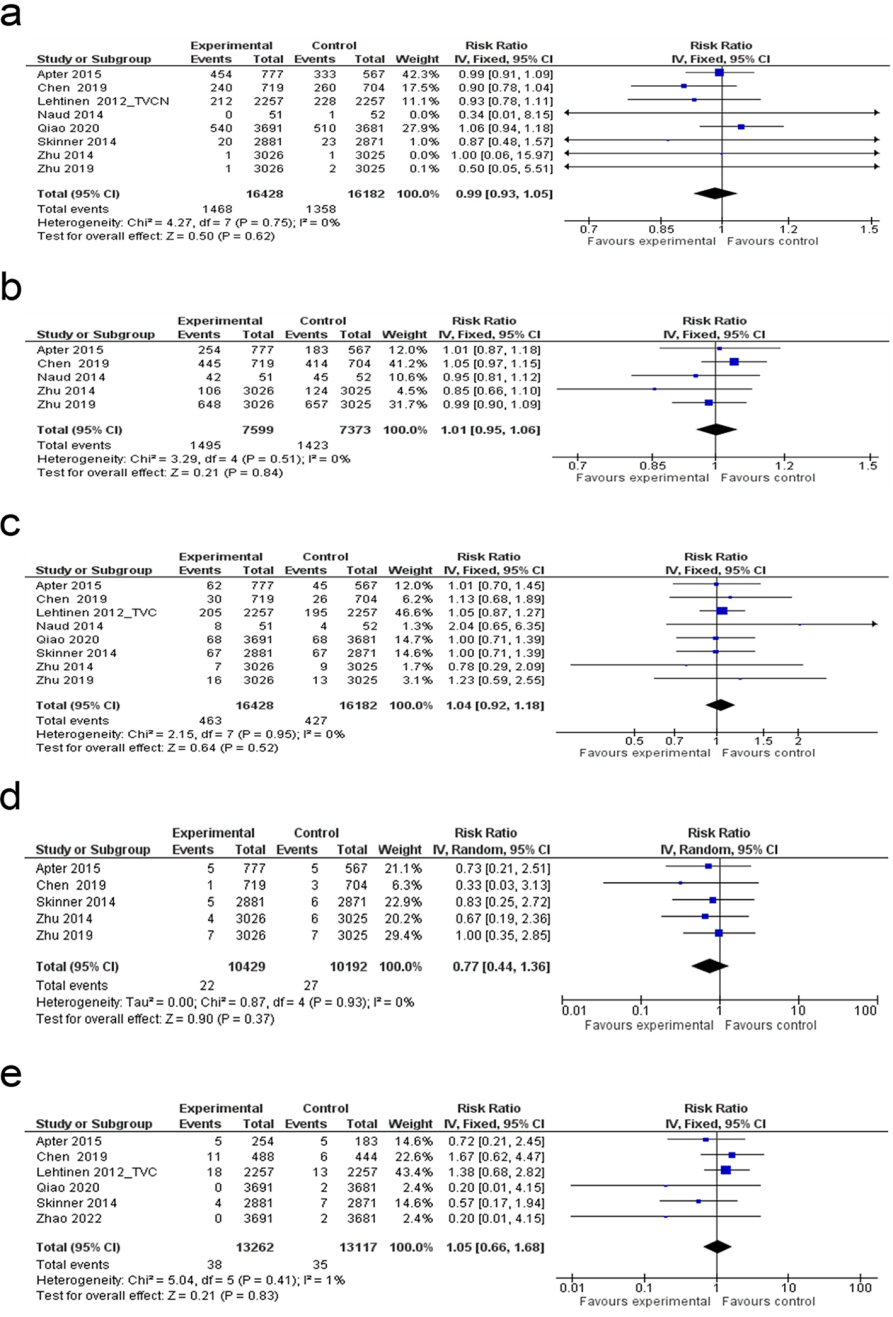
Forest plot of the **(a)** elective termination, **(b)** live birth, **(c)** spontaneous abortion **(d)** ectopic pregnancy and e) congenital anomalies rates.

**Figure 10:**
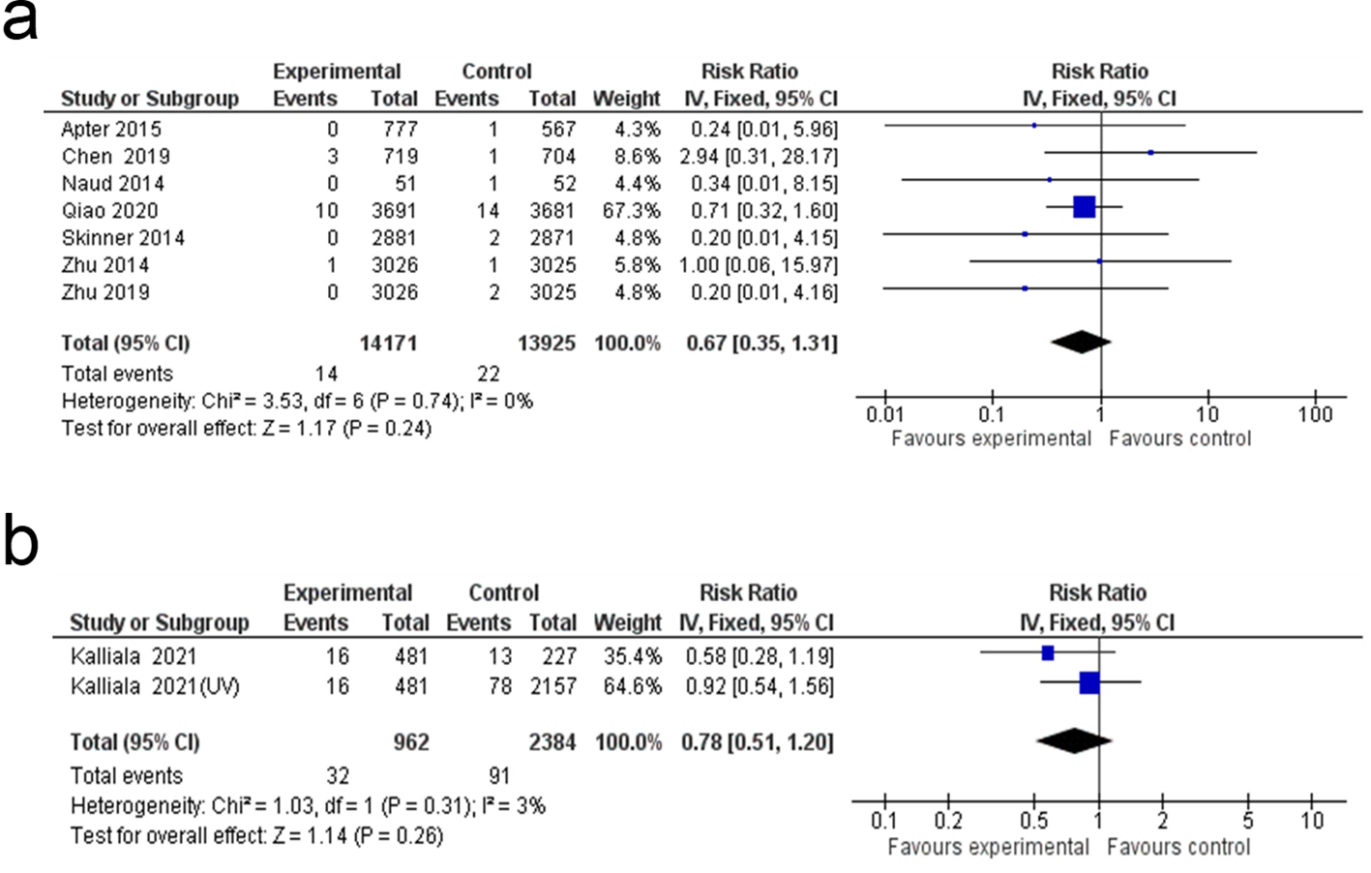
Forest plot of the **(a)** stillbirth and **(b)** preterm birth rate.

The meta-analysis showed no significant difference between the HPV vaccine and control groups across seven pregnancy-related outcomes. Elective termination rates were similar (RR: 0.99; 95% CI: 0.93 to 1.05; p = 0.62; I² = 0%). No significant differences were observed for live birth rates (RR: 1.01; 95% CI: 0.95 to 1.06; p = 0.84; I² = 0%) or spontaneous abortion (RR: 1.04; 95% CI: 0.92 to 1.18; p = 0.52; I² = 0%). Similarly, no significant differences were found in ectopic pregnancy (RR: 0.77; 95% CI: 0.44 to 1.36; p = 0.37; I² = 0%), congenital anomalies (RR: 1.05; 95% CI: 0.66 to 1.68; p = 0.83; I² = 1%), or stillbirth (RR: 0.67; 95% CI: 0.35 to 1.31; p = 0.24; I² = 0%). Lastly, preterm birth (PTB) rates showed no significant variation between groups (RR: 0.78; 95% CI: 0.51 to 1.20; p = 0.26; I² = 3%) (**Figures 9A–E**, **10A, B**).

### 3.25 Sensitivity analysis

The sensitivity analysis revealed that effect sizes remained largely stable following the exclusion of individual studies, with a few notable exceptions. For ASC-US, omitting *Lehtinen 2012_TVC* and *Lehtinen 2012_TVCN* adjusted the pooled RR from 0.31 (95% CI: 0.22–0.42; Z = 7.23; p < 0.00001) to 0.14 (95% CI: 0.06–0.35; Z = 4.25; p < 0.0001). Similarly, for LSIL, removing these studies modified the RR from 0.63 (95% CI: 0.49–0.81; Z = 3.66; p = 0.0003) to 0.14 (95% CI: 0.04–0.47; Z = 3.18; p = 0.001) (**Supplementary Figures S13-S14**).

### 3.26 Grading of evidence

According to the GRADE assessment, the quality of evidence varied across outcomes. HSIL, AIN, AIS, serious adverse events, elective termination, live birth, spontaneous abortion, ectopic pregnancy, congenital anomalies, stillbirth, and PTB were rated as high. CIN II, CIN III, LSIL, EGL, TCD4+, GMT6, GMT11, GMT16, injection-site adverse events, and systemic adverse events were classified as moderate. CIN I, persistent infections, incident infections, ASC-US, TCD8+, GMT18, and Grade 3 adverse events were categorized as low-quality (**Supplementary Table S6**).

## 4 Discussion

The results of this meta-analysis provide a comprehensive assessment of the efficacy, immunogenicity, and safety of prophylactic HPV vaccination across various clinical endpoints, reinforcing its role as a critical tool in HPV-associated disease prevention (**Supplementary Table S7**). Although seminal pre-2010 trials established the initial efficacy of prophylactic HPV vaccines, the present analysis emphasizes post-2010 randomized evidence that more accurately reflects contemporary vaccine formulations, standardized reporting practices, and implementation contexts relevant to current global vaccination strategies. The substantial risk reduction observed for CIN at all grades is consistent with prior meta-analyses, further supporting the long-term benefits of vaccination, particularly when administered before HPV exposure (59, 60). The most pronounced protective effect was observed for CIN I, where vaccination was associated with an 85% risk reduction. This finding aligns with prior reports indicating that early-stage lesions, which are primarily driven by transient HPV infections rather than established oncogenic transformation, are the most susceptible to vaccine-mediated protection (61, 62). The quadrivalent vaccine demonstrated the highest efficacy against CIN I, a trend potentially attributable to its broader antigenic spectrum, which includes HPV 6 and 11 in addition to high-risk HPV 16 and 18. While no statistically significant predictors of CIN I reduction were identified in meta-regression, the observation that younger individuals experienced the greatest benefit underscores the importance of early immunization, ideally before sexual debut (63).

For CIN II, the risk reduction following vaccination was slightly lower than that observed for CIN I, though still substantial at 80%. The bivalent vaccine exhibited the greatest protective effect against CIN II, likely due to its enhanced immunogenicity against HPV 16 and 18, which are responsible for the majority of high-grade lesions (64). The superior efficacy of the three-dose (0/1/6) regimen suggests that prolonged antigen exposure may enhance immunological memory, leading to more robust protection against progressive HPV-driven neoplasia. However, this apparent superiority should be interpreted in the context of the current evidence base, which is predominantly derived from three-dose schedules, while data on single-dose regimens remain limited and are largely restricted to immunogenicity outcomes with shorter clinical follow-up. The observed protective effect against CIN III, while significant, was more modest at 52%, consistent with prior studies demonstrating that high-grade lesions are less amenable to vaccine-mediated prevention (65). One explanation for this attenuation in efficacy is that CIN III often represents a more advanced stage of neoplastic progression, characterized by viral genome integration and oncogene overexpression (66). At this point, the HPV-driven carcinogenic process is largely mediated by the E6 and E7 oncoproteins, which promote immune evasion and cellular transformation by inactivating p53 and Rb (67). As vaccine-induced immunity primarily functions by preventing new infections rather than reversing established oncogenesis, its impact on CIN III may be inherently limited.

Beyond cervical lesions, this study demonstrates a strong vaccine effect in preventing persistent HPV 16/18 infections, with an 84% reduction in risk. The impact of vaccination on persistent infections was particularly notable in HIV-negative individuals, reinforcing prior findings that immunocompetent individuals mount more durable immune responses following immunization. This is supported by prior meta-analyses, which reported significantly lower antibody persistence in people living with HIV (PLHIV) (7, 68). The reduced immunogenicity in PLHIV likely stems from impaired dendritic cell function and suboptimal CD8^+^ T cell priming, both of which are critical for effective viral clearance (69). Notably, the vaccine was also highly effective in reducing incident HPV 16/18 infections, with a 75% risk reduction. While subgroup analysis suggested that the greatest benefit was seen in HIV-negative individuals, the overall heterogeneity of this outcome suggests that additional host and environmental factors may influence vaccine responsiveness.

The protective effect of HPV vaccination extended to cytological abnormalities, with significant reductions in ASC-US and LSIL. The risk of ASC-US was reduced by 69%, while LSIL incidence declined by 37%. These findings suggest that HPV vaccination confers broader cytological benefits beyond simply preventing high-grade lesions, likely by accelerating viral clearance and reducing the frequency of transient low-risk HPV infections (70). In contrast, the impact on HSIL was more modest, with an 18% reduction that approached statistical significance. This aligns with prior observations that HPV vaccines are most effective in preventing early, reversible stages of HPV-driven neoplasia rather than more advanced lesions (71).

The effect of HPV vaccination on extra-cervical lesions was also evaluated, revealing a 36% reduction in AIN and a 68% reduction in AIS. The strong protective effect against AIS, a lesion primarily associated with HPV 18, further supports the enhanced immunogenicity of the bivalent vaccine against HPV 16/18. The observed reduction in AIN is consistent with prior findings that HPV vaccines provide cross-protection against non-cervical HPV-associated lesions, particularly in high-risk populations such as men who have sex with men (MSM) (72, 73). However, the non-significant effect of vaccination on EGL suggests that the vaccine’s impact may be weaker against low-risk HPV types 6 and 11, which are the primary etiological agents of genital warts. This contrasts with prior meta-analyses that reported significant reductions in EGL risk following quadrivalent vaccination, underscoring the need for further research to clarify the vaccine’s impact on non-oncogenic HPV infections.

Immunologically, HPV vaccination elicited robust humoral responses, with significant increases in anti-HPV6, HPV11, HPV16, and HPV18 antibody titers (74). The greatest fold increase was observed for anti-HPV16 antibodies, which rose 3.09-fold following vaccination. Although, the same fold increase in anti-HPV18 antibodies (3.10-fold) is opposite to prior reports indicating that HPV 18 elicits a weaker neutralizing antibody response than HPV 16. Structural differences in the L1 capsid protein between HPV 16 and HPV 18 may contribute to this discrepancy by influencing antigen presentation and epitope availability (75). The absence of a statistically significant effect on tissue-resident CD8^+^ T cells suggests that vaccine-mediated immunity is predominantly humoral rather than cell-mediated, though the observed reduction in tissue CD4^+^ T cells raises intriguing questions regarding potential immunomodulatory effects (76). Given that HPV establishes persistent infection in the basal epithelial layer, where it evades immune detection, the potential for vaccine-induced alterations in mucosal immune composition warrants further exploration.

The safety profile of HPV vaccines remains favorable, with no significant increase in serious adverse events or systemic adverse events observed. However, a 26% increase in injection-site reactions was noted, particularly with the quadrivalent and nonavalent vaccines. This is consistent with prior reports attributing increased reactogenicity to the aluminum-based adjuvants used in these formulations, which enhance antigen uptake but also provoke localized inflammation (77). Also, the consistency of safety estimates across outcomes suggests that the occurrence of severe, systemic, and injection-site adverse events is largely independent of surveillance duration. For prophylactic vaccines such as HPV, clinically relevant adverse reactions tend to be temporally proximate to immunization and are primarily driven by vaccine formulation and host reactogenicity rather than delayed biological processes. As a result, extending follow-up periods may increase the opportunity to observe rare events but is unlikely to substantially alter the overall pattern or frequency of common or severe adverse reactions. Importantly, pregnancy outcomes were not adversely affected by vaccination, with no significant differences in rates of elective termination, live birth, spontaneous abortion, ectopic pregnancy, congenital anomalies, stillbirth, or PTB between vaccinated and unvaccinated groups. These findings provide strong reassurance regarding the safety of HPV vaccination in reproductive-age individuals (78).

An important consideration is that, sex-specific immune and clinical responses to vaccination are biologically relevant, as sex hormones, immune activation thresholds, and exposure patterns may influence both vaccine efficacy and reactogenicity. In this study, sex-stratified analyses were performed where data permitted and suggested broadly comparable vaccine effectiveness across males and females for major clinical endpoints, including CIN-related outcomes and non-cervical lesions. For certain safety outcomes, notably injection-site adverse events, sex-based subgroup differences were observed; however, their interpretation warrants caution. Across multiple endpoints, sex-stratified inference was constrained by heterogeneous reporting practices, with many trials enrolling female-only populations or not reporting sex-specific data, and relatively few male-only studies contributing to pooled estimates. Consequently, while our analyses provide supportive evidence for gender-neutral vaccination strategies, definitive conclusions regarding sex-specific differences in vaccine responses remain limited by the structure and reporting of the available evidence base.

Despite the strong evidence supporting HPV vaccination, some limitations must be acknowledged. The substantial heterogeneity observed in multiple analyses—ranging from moderate to high across efficacy and infection-related outcomes (I² approximately 60–95%) and frequently exceeding 90% for immunogenicity endpoints—suggests that factors such as age at vaccination, prior HPV exposure, vaccine formulation, dosing schedules, and follow-up duration may modulate vaccine effectiveness and contribute to between-study variability. Although subgroup and meta-regression analyses explored these modifiers, residual heterogeneity indicates that unmeasured clinical and methodological differences across trials likely persist. Importantly, this heterogeneity appears to be largely statistical rather than contradictory in direction, as effect estimates were generally consistent and sensitivity analyses did not materially alter the pooled results. Additionally, the presence of publication bias in certain outcomes indicates that smaller studies reporting lower vaccine efficacy may be underrepresented, potentially inflating pooled estimates despite the robustness observed in trim-and-fill and sensitivity analyses. Another important consideration is the potential for language and regional bias. In addition, the prophylactic efficacy of HPV vaccination against specific non-cervical outcomes, including anogenital warts and vulvar or vaginal diseases, could not be comprehensively evaluated due to the limited number of studies reporting these endpoints and the heterogeneity in outcome definitions across trials. Additionally, potential associations between prophylactic HPV vaccination and autoimmune diseases could not be evaluated, as autoimmune outcomes were not prespecified and were rarely or inconsistently reported across the included RCTs. While comprehensive database searches were conducted, the predominance of English-language publications and the substantial contribution of trials conducted in specific geographic regions—particularly East Asia—may limit the generalizability of the findings to broader global populations with differing epidemiological, socioeconomic, and healthcare contexts. Moreover, by restricting inclusion to RCTs, this review may underrepresent real-world vaccine effectiveness observed in large observational and population-based studies, potentially limiting applicability to routine immunization settings. Furthermore, industry sponsorship in several included RCTs cannot be fully excluded as a source of bias, although most studies adhered to rigorous methodological standards and regulatory oversight. In addition, while nonavalent and multi-dose vaccination strategies offer maximal immunological and clinical protection, their cost-effectiveness and equitable implementation in low-resource settings warrant careful consideration, particularly in regions with constrained healthcare budgets and limited vaccine access. Moreover, although a small number of studies evaluating single-dose and two-dose regimens were included, the available evidence was predominantly derived from three-dose schedules, and single-dose data were largely limited to immunogenicity outcomes with shorter clinical follow-up. Future research should prioritize long-term cohort studies to assess the durability of vaccine-induced immunity beyond 10 years, as well as RCTs comparing single-dose versus multi-dose regimens. Further investigation into vaccine efficacy in immunocompromised populations, particularly HIV positivity, is warranted to determine whether modified dosing schedules or booster doses may be necessary. Mechanistic studies exploring the immunological basis of vaccine protection, including the role of HPV-specific memory B cells and regulatory T cell interactions, could further refine our understanding of HPV vaccine biology.

Based on the findings of this meta-analysis, the nonavalent vaccine appears to offer the most comprehensive protection for widespread immunization, with a three-dose (0/1/6 month) regimen providing maximal benefit, particularly when administered before sexual debut. However, this conclusion should be interpreted in the context of variable certainty across outcomes, including low-quality evidence for certain endpoints, particularly immunogenicity and subgroup-based estimates, highlighting the importance of context-specific implementation and ongoing evidence generation. Expanding gender-neutral vaccination programs remains a critical public health priority, as achieving high population coverage is essential to reducing the global burden of HPV-associated malignancies.

## 5 Conclusion

This meta-analysis demonstrates the strong efficacy of prophylactic HPV vaccination in reducing HPV infections, CIN I–III, AIS, and AIN, with a consistently favorable safety profile. The nonavalent vaccine, particularly in a three-dose (0/1/6) regimen, offers the broadest protection, with the greatest benefit observed in younger populations. Robust humoral immune responses were induced, alongside no increased risk of serious adverse events or adverse pregnancy outcomes. These findings support early, gender-neutral HPV vaccination as a key public health strategy, while highlighting the need for continued high-quality and long-term studies to strengthen evidence for selected outcomes.

## Data Availability

The original contributions presented in the study are included in the article/**Supplementary Material**. Further inquiries can be directed to the corresponding authors.
