# Supplementary material for: Comprehensive evaluation of prophylactic HPV vaccines: a systematic review and meta-analysis of efficacy, safety, and immunogenicity in males and females
Source: Front Immunol. 2026 Jan 13;16:1747082. doi: 10.3389/fimmu.2025.1747082 (PMC12835409; doi:10.3389/fimmu.2025.1747082)
Supplement: Supplementary file 1 [file Table1.docx]

**Table S1.** Definitions of Outcomes for assessment of the efficacy, safety, and immunogenicity of prophylactic HPV vaccines.

| Outcome | Definition |
| --- | --- |
| CIN (1)-HPV16-18 | Mild dysplasia of cervical cells caused by HPV16/18 infection, considered a low-grade precancerous lesion. |
| CIN (2)-HPV16-18 | Moderate cervical dysplasia associated with HPV16/18, with a higher risk of progression to cervical cancer. |
| CIN (3)-HPV16-18 | Severe cervical dysplasia with a high likelihood of progressing to invasive cervical cancer. |
| Persistent infections (HPV16-18) | Persistent HPV16/18 infection was defined as the detection of HPV16 and/or HPV18 DNA in cervical samples at two or more consecutive visits spanning at least 12 months, as assessed by polymerase chain reaction (PCR)–based HPV DNA testing, in accordance with definitions used in the included randomized controlled trials. |
| Incident infections (HPV16-18) | Incident HPV16/18 infection was defined as a newly detected HPV16 and/or HPV18 DNA–positive result during follow-up in participants who were HPV16/18 DNA–negative at baseline, measured using PCR-based HPV DNA assays. |
| ASC-US | Atypical squamous cells of undetermined significance; minor cervical cell abnormalities that may be caused by HPV infection. |
| HSIL | High-grade squamous intraepithelial lesion; a significant abnormality in cervical cells with a high risk of progression to cancer. |
| LSIL | Low-grade squamous intraepithelial lesion; mild cervical cell changes caused by transient HPV infection, often resolving spontaneously. |
| AIN | Anal intraepithelial neoplasia; a precancerous lesion in the anal epithelium caused by persistent HPV infection. |
| AIS | Adenocarcinoma in situ; a non-invasive glandular lesion of the cervix that can develop into adenocarcinoma if untreated. |
| EGL | External genital lesions; HPV-induced lesions affecting external genitalia, including genital warts and precancerous changes. |
| TCD4+ | Tissue-resident CD4+ T cells; indicators of local immune response to HPV infection. |
| TCD8+ | Tissue-resident CD8+ T cells; cytotoxic immune cells involved in clearing HPV-infected epithelial cells. |
| GMT 6 | Geometric mean titer of anti-HPV6 antibodies, measuring the immune response to HPV6 post-vaccination. |
| GMT 11 | Geometric mean titer of anti-HPV11 antibodies, indicating vaccine-induced immunity against HPV11. |
| GMT 16 | Geometric mean titer of anti-HPV16 antibodies, linked to vaccine protection against HPV16-driven malignancies. |
| GMT 18 | Geometric mean titer of anti-HPV18 antibodies, reflecting vaccine efficacy in preventing HPV18-related infections. |
| AEs (grade 3) | Severe adverse events of grade 3 or higher, including systemic reactions requiring medical intervention. |
| Serious AEs | Life-threatening or disabling events occurring after vaccination, evaluated for safety assessment. |
| Injection-site AE | Localized pain, swelling, or redness at the injection site post-vaccination, commonly observed side effects. |
| Systemic AE | Generalized adverse effects such as fever, fatigue, or headache following HPV vaccination. |
| Elective Termination | Voluntary termination of pregnancy, analyzed in relation to HPV vaccination safety. |
| Live Birth | The proportion of pregnancies resulting in a live birth, assessed in vaccinated versus unvaccinated individuals. |
| Spontaneous Abortion | Pregnancy loss occurring naturally before 20 weeks of gestation, evaluated for vaccine safety. |
| Ectopic Pregnancy | Implantation of a fertilized egg outside the uterus, analyzed for potential association with HPV vaccination. |
| Congenital Anomalies | Birth defects observed in infants born to vaccinated mothers, assessed for vaccine-related risks. |
| Stillbirth | Fetal death occurring after 20 weeks of pregnancy, studied in relation to HPV vaccine exposure. |
| Preterm Birth (PTB) | Delivery occurring before 37 weeks of gestation, analyzed for potential correlation with HPV vaccination. |

**Table S2.** Characteristics of the studies included.

| Author & Year (Clinical Trial Code) | Population | Intervention Group | Control Group | Vaccine Characteristics in Test Group | Country/mean age | Intervention/ Follow-up Duration in Test Group (months) |
| --- | --- | --- | --- | --- | --- | --- |
| Apter, 2015 (NCT001226810) | Young women from the general population | Bivalent HPV^†^ AS04-adjuvanted vaccine (Cervarix) | Hepatitis A vaccine | 3 doses at months 0, 1, and 6 | Multinational/20 | 6/66 |
| Apter, 2015-1 (NCT001226810) TVCN: total vaccinated cohort – naïve | Young women, HPV-naïve with normal cervical cytology | Bivalent HPV AS04-adjuvanted vaccine (Cervarix) | Hepatitis A vaccine | 3 doses at months 0, 1, and 6 | Multinational/19.9 | 6/66 |
| Apter, 2015-2 (NCT001226810) TVC: total vaccinated cohort | Young women from the general population | Bivalent HPV AS04-adjuvanted vaccine (Cervarix) | Hepatitis A vaccine | 3 doses at months 0, 1, and 6 | Multinational/20 | 6/66 |
| Apter, 2015-1-12m^†^ (NCT001226810) | Young women, HPV-naïve with normal cervical cytology | Bivalent HPV AS04-adjuvanted vaccine (Cervarix) | Hepatitis A vaccine | 3 doses at months 0, 1, and 6 | Multinational/19.9 | 6/66 |
| Apter, 2015-1-6m (NCT001226810) | Young women, HPV-naïve with normal cervical cytology | Bivalent HPV AS04-adjuvanted vaccine (Cervarix) | Hepatitis A vaccine | 3 doses at months 0, 1, and 6 | Multinational/19.9 | 6/66 |
| Apter, 2015-2-6m (NCT001226810) | Young women from the general population | Bivalent HPV AS04-adjuvanted vaccine (Cervarix) | Hepatitis A vaccine | 3 doses at months 0, 1, and 6 | Multinational//20 | 6/66 |
| Apter, 2015-2-12m (NCT001226810) | Young women from the general population | Bivalent HPV AS04-adjuvanted vaccine (Cervarix) | Hepatitis A vaccine | 3 doses at months 0, 1, and 6 | Multinational/20 | 6/66 |
| Apter, 2015-3-6m (NCT001226810) TVCP: total vaccinated cohort – positive | Young women with prior HPV exposure | Bivalent HPV AS04-adjuvanted vaccine (Cervarix) | Hepatitis A vaccine | 3 doses at months 0, 1, and 6 | Multinational/20 | 6/66 |
| Apter, 2015-3-12m (NCT001226810) | Young women with prior HPV exposure | Bivalent HPV AS04-adjuvanted vaccine (Cervarix) | Hepatitis A vaccine | 3 doses at months 0, 1, and 6 | Multinational/20 | 6/66 |
| Chen 2019 (NCT00834106) | Chinese women from the general population | Quadrivalent HPV vaccine | Placebo | 3 doses at months 0, 1, and 6 | China/28.7 | 6/90 |
| De Carvalho 2010 (NCT00518336) | Young women from the general population | Bivalent HPV AS04-adjuvanted vaccine (Cervarix) | Placebo | 3 doses at months 0, 1, and 6 | Brazil/20.4 | 6/87.6 |
| De Carvalho 2010 (1.5y)  y: year | Young women from the general population | Bivalent HPV AS04-adjuvanted vaccine (Cervarix) | Placebo | 3 doses at months 0, 1, and 6 | Brazil/20.4 | 6/87.6 |
| De Carvalho 2010 (12m) | Young women from the general population | Bivalent HPV AS04-adjuvanted vaccine (Cervarix) | Placebo | 3 doses at months 0, 1, and 6 | Brazil/20.4 | 6/87.6 |
| De Carvalho 2010 (6.4y) | Young women from the general population | Bivalent HPV AS04-adjuvanted vaccine (Cervarix) | Placebo | 3 doses at months 0, 1, and 6 | Brazil/20.4 | 6/87.6 |
| De Carvalho 2010 (6m) | Young women from the general population | Bivalent HPV AS04-adjuvanted vaccine (Cervarix) | Placebo | 3 doses at months 0, 1, and 6 | Brazil/20.4 | 6/87.6 |
| De Carvalho 2010 (7.3y) | Young women from the general population | Bivalent HPV AS04-adjuvanted vaccine (Cervarix) | Placebo | 3 doses at months 0, 1, and 6 | Brazil/20.4 | 6/87.6 |
| Denny 2013 (NCT00779766) | Young women from the general population, including HIV-positive and HIV-negative participants | Bivalent HPV AS04-adjuvanted vaccine (Cervarix) | placebo | 3 doses at months 0, 1, and 6 | South Africa/22 | 6/12 |
| Denny 2013 (NCT00779766)  HP: HIV positive | Young HIV-positive women from the general population | Bivalent HPV AS04-adjuvanted vaccine (Cervarix) | placebo | 3 doses at months 0, 1, and 6 | South Africa/22.1 | 6/12 |
| Denny 2013 (NCT00779766) HN: HIV negative | Young HIV-negative women from the general population | Bivalent HPV AS04-adjuvanted vaccine (Cervarix) | placebo | 3 doses at months 0, 1, and 6 | South Africa/21.1 | 6/12 |
| Garland 2015 (NCT01047345) | Young women previously vaccinated with quadrivalent HPV vaccine | 9-valent HPV vaccine | Saline placebo | 3 doses at months 0, 2, and 6 | Multinational/19 | 6/7 |
| Garland2015(2M) (NCT01047345) | Young women previously vaccinated with quadrivalent HPV vaccine | 9-valent HPV vaccine | Saline placebo | 3 doses at months 0, 2, and 6 | Multinational/19 | 6/7 |
| Garland2015(7M) (NCT01047345) | Young women previously vaccinated with quadrivalent HPV vaccine | 9-valent HPV vaccine | Saline placebo | 3 doses at months 0, 2, and 6 | Multinational/19 | 6/7 |
| Garland 2016 | Young women from the general population | HPV AS04-adjuvanted vaccine (Cervarix) | Hepatitis A vaccine | 3 doses at months 0, 1, and 6 | Multinational/20 | 6/39 |
| Gilson, 2020(IM) (NCT001226810)  IM: Imiquimod | Adults with anogenital warts; majority male (~66%), including heterosexuals and MSM; small proportion HIV-positive (~2%) | Quadrivalent HPV vaccine | IM plus placebo | 3 doses at weeks 0, 8, and 24 | UK (England & Wales)/31 | 16/48 |
| Gilson 2020(PD) (NCT001226810)  PD: podophyllotoxin | Adults with anogenital warts, predominantly male adults | Quadrivalent HPV vaccine | Placebo | 3 doses at weeks 0, 8, and 24 | UK (England & Wales)/31 | 4/12 |
| Goldstone 2013 (ITT) (NCT00090285), ITT: intention-to-treat | Young men, including heterosexual men and men who have sex with men | HPV AS04-adjuvanted vaccine | Placebo | 3 doses at months 0, 2, and 6 | Multinational/20.5 | 6/36 |
| Goldstone (naive) 2013 (NCT00090285) | Young men, HPV-naïve at baseline, including heterosexual men and men who have sex with men | HPVAS04-adjuvanted vaccine | Placebo | 2 doses at months 0, 2, and 6 | Multinational/ 20.5 | 6/36 |
| Goldstone, 2022 (1) (V501–020–21) | Young men, including heterosexual men and men who have sex with men | HPVAS04-adjuvanted vaccine | Placebo | 3 doses at months 0, 2, and 6 | Multinational20.5 | 6/120 |
| Goldstone2022 (2) (V501–020–21) | Young men, including heterosexual men and men who have sex with men | HPVAS04-adjuvanted vaccine | Placebo | 3 doses at months 0, 2, and 6 | Multinational/20.5 | 6/120 |
| Herrero, 2011 (NCT00128661) ATP: according-to-protocol | Young women (18–25 years) from the general population | HPVAS04-adjuvanted vaccine | Hepatitis A vaccine | 3 doses at 0, 1, and 6 months | Costa Rica/NR | 6/50.4 |
| Herrero 2011(ITT) (NCT00128661) ITT: intention-to-treat | Young women (18–25 years) from the general population | HPVAS04-adjuvanted vaccine | Hepatitis A vaccine | 3 doses at 0, 1, and 6 months | Costa Rica/NR | 6/50.4 |
| Hidalgo-Tenorio 2021(ISRCTN14732216) | HIV-positive MSM | Quadrivalent HPV vaccine (Gardasil) | Placebo | 3 doses at months 0, 2, and 6 | Spain/ 38.9 | 6/48 |
| Hidalgo-Tenorio2021 (1) (ISRCTN14732216) | HIV-positive MSM | Quadrivalent HPV vaccine (Gardasil) | Placebo | 3 doses at months 0, 2, and 6 | Spain/ 38.9 | 6/48 |
| Hidalgo-Tenorio2021 (2) (ISRCTN14732216) | HIV-positive MSM | Quadrivalent HPV vaccine (Gardasil) | Placebo | 3 doses at months 0, 2, and 6 | Spain/ 38.9 | 6/48 |
| Hidalgo-Tenorio2021 (3) (ISRCTN14732216) | HIV-positive MSM | Quadrivalent HPV vaccine (Gardasil) | Placebo | 3 doses at months 0, 2, and 6 | Spain/ 38.9 | 6/48 |
| Hidalgo-Tenorio2021 (4) (ISRCTN14732216) | HIV-positive MSM | Quadrivalent HPV vaccine (Gardasil) | Placebo | 3 doses at months 0, 2, and 6 | Spain/ 38.9 | 6/48 |
| Hidalgo-Tenorio2021 (5) (ISRCTN14732216) | HIV-positive MSM | Quadrivalent HPV vaccine (Gardasil) | Placebo | 3 doses at months 0, 2, and 6 | Spain/ 38.9 | 6/48 |
| Hildesheim 2014 (NCT00128661) | Young women (18–25 years) from the general population | Bivalent HPV vaccine | Hepatitis A vaccine | 3 doses at 0, 1, and 6 months | Costa Rica/NR | 6/48 |
| Hildesheim 2014 (1) (NCT00128661) | Young women (18–25 years) from the general population | Bivalent HPV vaccine | Hepatitis A vaccine | 3 doses at 0, 1, and 6 months | Costa Rica/NR | 6/48 |
| Hildesheim 2014 (2) (NCT00128661) | Young women (18–25 years) from the general population | Bivalent HPV vaccine | Hepatitis A vaccine | 3 doses at 0, 1, and 6 months | Costa Rica/NR | 6/48 |
| Hildesheim 2014 (3) (NCT00128661) | Young women (18–25 years) from the general population | Bivalent HPV vaccine | Hepatitis A vaccine | 3 doses at 0, 1, and 6 months | Costa Rica/NR | 6/48 |
| Hildesheim 2014 (4) (NCT00128661) | Young women (18–25 years) from the general population | Bivalent HPV vaccine | Hepatitis A vaccine | 3 doses at 0, 1, and 6 months | Costa Rica/NR | 6/48 |
| Hillman 2012 (NCT00090285) | Males from the general population, including MSM | Quadrivalent HPV vaccine (Gardasil) | AAHS-containing placebo N=999 | 3 doses at months 0, 1, and 6 | Multinational/ 21.2 | 6/36 |
| Hu 2021 (NCT00779766) | Young women with baseline high-risk HPV infection | Quadrivalent HPV vaccine (Gardasil) | AAHS-containing placebo N=1000 | 3 doses at months 0, 1, and 6 | China /23 | 6/36 |
| Hu 2021(1) (NCT00779766) | Young women with baseline high-risk HPV infection | Bivalent HPV AS04-adjuvanted vaccine (Cervarix) | Aluminum hydroxide placebo | 3 doses at months 0, 1, and 6 | China /23 | 6/72 |
| Hu 2021(2) (NCT00779766) | Young women with baseline high-risk HPV infection | Bivalent HPV AS04-adjuvanted vaccine (Cervarix) | Aluminum hydroxide placebo | 3 doses at months 0, 1, and 6 | China /23 | 6/72 |
| Hu 2021(3) (NCT00779766) | Young women with baseline high-risk HPV infection | Bivalent HPV AS04-adjuvanted vaccine (Cervarix) | Aluminum hydroxide placebo | 3 doses at months 0, 1, and 6 | China /23 | 6/72 |
| Hu 2021(4) (NCT00779766) | Young women with baseline high-risk HPV infection | Bivalent HPV AS04-adjuvanted vaccine (Cervarix) | Aluminum hydroxide placebo | 3 doses at months 0, 1, and 6 | China /23 | 6/72 |
| Hu 2021(5) (NCT00779766) | Young women with baseline high-risk HPV infection | Bivalent HPV AS04-adjuvanted vaccine (Cervarix) | Aluminum hydroxide placebo | 3 doses at months 0, 1, and 6 | China /23 | 6/72 |
| Hu 2021(6) (NCT00779766) | Young women with baseline high-risk HPV infection | Bivalent HPV AS04-adjuvanted vaccine (Cervarix) | Aluminum hydroxide placebo | 3 doses at months 0, 1, and 6 | China /23 | 6/72 |
| Hu 2021-1(12m) (NCT00779766) | Young women with baseline high-risk HPV infection | Bivalent HPV AS04-adjuvanted vaccine (Cervarix) | Aluminum hydroxide placebo | 3 doses at months 0, 1, and 6 | China /23 | 6/72 |
| Hu 2021-1(6m) (NCT00779766) | Young women with baseline high-risk HPV infection | Bivalent HPV AS04-adjuvanted vaccine (Cervarix) | Aluminum hydroxide placebo | 3 doses at months 0, 1, and 6 | China /23 | 6/72 |
| Hu2021-2(12m) (NCT00779766) | Young women with baseline high-risk HPV infection | Bivalent HPV AS04-adjuvanted vaccine (Cervarix) | Aluminum hydroxide placebo | 3 doses at months 0, 1, and 6 | China /23 | 6/72 |
| Hu 2021-2(6m) (NCT00779766) | Young women with baseline high-risk HPV infection | Bivalent HPV AS04-adjuvanted vaccine (Cervarix) | Aluminum hydroxide placebo | 3 doses at months 0, 1, and 6 | China /23 | 6/72 |
| Hu 2021-3(12m) (NCT00779766) | Young women with baseline high-risk HPV infection | Bivalent HPV AS04-adjuvanted vaccine (Cervarix) | Aluminum hydroxide placebo | 3 doses at months 0, 1, and 6 | China /23 | 6/72 |
| Hu 2021-3(6m) (NCT00779766) | Young women with baseline high-risk HPV infection | Bivalent HPV AS04-adjuvanted vaccine (Cervarix) | Aluminum hydroxide placebo | 3 doses at months 0, 1, and 6 | China /23 | 6/72 |
| Hu 2021-4(12m) (NCT00779766) | Young women with baseline high-risk HPV infection | Bivalent HPV AS04-adjuvanted vaccine (Cervarix) | Aluminum hydroxide placebo | 3 doses at 0, 1, and 6 months | China /23 | 6/72 |
| Hu 2021-4(6m) (NCT00779766) | Young women with baseline high-risk HPV infection | Bivalent HPV AS04-adjuvanted vaccine (Cervarix) | Aluminum hydroxide placebo | 3 doses at months 0, 1, and 6 | China /23 | 6/72 |
| Hu 2021-5(12m) (NCT00779766) | Young women with baseline high-risk HPV infection | Bivalent HPV AS04-adjuvanted vaccine (Cervarix) | Aluminum hydroxide placebo | 3 doses at months 0, 1, and 6 | China /23 | 6/72 |
| Hu 2021-5(6m) (NCT00779766) | Young women with baseline high-risk HPV infection | Bivalent HPV AS04-adjuvanted vaccine (Cervarix) | Aluminum hydroxide placebo | 3 doses at months 0, 1, and 6 | China /23 | 6/72 |
| Hu 2021-6(12m) (NCT00779766) | Young women with baseline high-risk HPV infection | Bivalent HPV AS04-adjuvanted vaccine (Cervarix) | Aluminum hydroxide placebo | 3 doses at months 0, 1, and 6 | China /23 | 6/72 |
| Hu 2021-6(6m) (NCT00779766) | Young women with baseline high-risk HPV infection | Bivalent HPV AS04-adjuvanted vaccine (Cervarix) | Aluminum hydroxide placebo | 3 doses at months 0, 1, and 6 | China /23 | 6/72 |
| Hu, 2023 (NCT03935204) | Healthy women aged 18–45 years | Bivalent HPV AS04-adjuvanted vaccine (Cervarix) | Placebo | 3 doses at months 0, 1, and 6 | China/NR | 6/7 |
| Kalliala (2021) (NCT00534638) | Women from population-based birth cohorts | Bivalent HPV AS04-adjuvanted vaccine (Cervarix) | HBV Vaccine - Engerix® B | 3 doses at months 0, 1, and 6 | Finland/NR | 6/84 |
| Kalliala 2021 (UV) (NCT00534638)  UV: unvaccinated | Women from population-based birth cohorts | Bivalent HPV AS04-adjuvanted vaccine (Cervarix) | HBV Vaccine - Engerix® B | 4 doses at months 0, 1, and 6 | Finland/NR | 6/84 |
| Kim 2010 (NCT00290277) | Healthy girls | Bivalent HPV AS04-adjuvanted vaccine (Cervarix) | Hepatitis A vaccine | 3 doses at months 0, 1, and 6 | Korea/11.9 | 6/7 |
| Kim, 2011 (NCT00485732) | Healthy women | Bivalent HPV AS04-adjuvanted vaccine (Cervarix) | Aluminum hydroxide placebo | 3doses at months 0, 1, and 6 | Korea/22 | 6/7 |
| Kim 2011 (SN) (NCT00485732)  SN: seronegative | Seronegative women for HPV-16 and HPV-18 at baseline | Bivalent HPV AS04-adjuvanted vaccine (Cervarix) | Aluminum hydroxide placebo | 3 doses at months 0, 1, and 6 | Korea/22 | 6/7 |
| Kim 2011 (SP) (NCT00485732)  SP: seropositive | Seropositive women for HPV-16 and/or HPV-18 at baseline | Bivalent HPV AS04-adjuvanted vaccine (Cervarix) | Aluminum hydroxide placebo | 3 doses at months 0, 1, and 6 | Korea/22 | 6/7 |
| Kim 2011(tot) (NCT00485732)  Tot: total | Healthy women | Bivalent HPV AS04-adjuvanted vaccine (Cervarix) | Aluminum hydroxide placebo | 3 doses at months 0, 1, and 6 | Korea/22 | 6/7 |
| Konno 2010 (1) (NCT00316693) | Women aged 20–25 years | Bivalent HPV AS04-adjuvanted vaccine | Hepatitis A vaccine | 3 doses at months 0, 1, and 6 | Japan/NR | 6/24 |
| Konno 2010 (2) (NCT00316693) | Women aged 20–25 years | Bivalent HPV AS04-adjuvanted vaccine | Hepatitis A vaccine | 3 doses at months 0, 1, and 6 | Japan/NR | 6/24 |
| Kreimer 2011 (NCT00128661) | Young women (18–25 years) from the general population | Bivalent HPV AS04-adjuvanted vaccine (Cervarix) | Hepatitis A vaccine | 3 doses at months 0, 1, and 6 | Costa Rica/NR | 6/48 |
| Kreimer 2020 (1) (NCT00128661) | Young women (18–25 years) from the general population | Bivalent HPV vaccine (Cervarix) | Hepatitis A vaccine | Single dose | Costa Rica/NR | 1/136 |
| Kreimer 2020 (2) (NCT00128661) | Young women (18–25 years) from the general population | Bivalent HPV vaccine (Cervarix) | Hepatitis A vaccine | Two doses at 0 and 6 months | Costa Rica/NR | 6/76 |
| Kreimer 2020 (3) (NCT00128661) | Young women (18–25 years) from the general population | Bivalent HPV vaccine (Cervarix) | Hepatitis A vaccine | 3 doses at months 0, 1, and 6 | Costa Rica/NR | 6/76 |
| Lehtinen 2012TVC (PATRICIA Trial) TVC: total vaccinated cohort | Young women from the general population (15–25 years), irrespective of baseline HPV DNA, serostatus, or cytology | Bivalent HPV AS04-adjuvanted vaccine (CervarixÂ) | Hepatitis A vaccine | 3 doses at months 0, 1, and 6 | Multinational/NR | 6/48 |
| Lehtinen 2012TVCN (PATRICIA Trial) TVCN: total vaccinated cohort-naive | Young women from the general population (15–25 years), HPV-naïve at baseline (DNA-negative for oncogenic HPV, seronegative for HPV-16/18, normal cytology) | Bivalent HPV AS04-adjuvanted vaccine (CervarixÂ) | Hepatitis A vaccine | 3 doses at months 0, 1, and 6 | Multinational/NR | 6/48 |
| Lehtinen 2016 (NCT00122681) | Adolescents (girls and boys) aged 12–15 years | Bivalent HPV AS04-adjuvanted vaccine (CervarixÂ) | Hepatitis A vaccine | 3 doses at months 0, 1, and 6 | Finland/NR | 6/48 |
| Lehtinen 2019 (NCT00534638) | Women from population-based cohorts; vaccinated at ages 16–17 and age-aligned controls | Bivalent HPV AS04-adjuvanted vaccine (CervarixÂ) | Hepatitis B vaccine (Engerix B) | 3 doses at months 0, 1, and 6 | Finland//NR | 6/72 |
| Levin 2010 (NCT00339040) | HIV-infected children from the general population | HPV quadrivalent vaccine (Gardasil Â) | Placebo | 3 doses at 0,1, and 6 months | USA/10 | 6/7 |
| Li 2012 | Healthy males and females from the general population | Quadrivalent HPV vaccine | Placebo | 3 doses at 0, 2, and 6 months | China/24.6 | 6/7 |
| Mikamo 2019 (NCT01862874) | Young Japanese men from the general population | Quadrivalent HPV vaccine | Placebo | 3 doses at months 0, 1, and 6 | Japan/22.6 | 6/36 |
| Mo 2022 (NCT02405520) A | Healthy adult men and women from the general population | Bivalent HPV vaccine, (30µg) | Placebo | 3 doses at months 0, 1, and 6 | China/ 35.7 | 6/7 |
| Mo 2022 (NCT02405520) B | Healthy adult men and women from the general population | Bivalent HPV vaccine, (60µg) | Placebo | 3 doses at months 0, 1, and 6 | China/ 35.7 | 6/7 |
| Mo 2022 (NCT02405520) C | Healthy adult men and women from the general population | Bivalent HPV vaccine, (90µg) | Placebo | 3 doses at months 0, 1, and 6 | China/ 35.7 | 6/7 |
| Moreira 2011 (NCT00090285) | Healthy males from the general population | Quadrivalent HPV Vaccine | Placebo | 3 doses at months 0, 1, and 6 | Multinational/20.3 | 6/34.8 |
| Naud 2014 (NCT00518336) | Young women from the general population | Bivalent HPV vaccine | Placebo | 3 doses at 0, 1, and 6 months | Brazil/19.9 | 6/113 |
| Palefsky 2011 (NCT00090285) | Young HIV-negative men who have sex with men from the general population | Quadrivalent vaccine (Gardasil) | Placebo | 3 doses at months 0, 2, and 6 | Multinational/22 | 6/36 |
| Palefsky 2011 (1) PPP (NCT00090285) per-protocol population | Young HIV-negative men who have sex with men from the general population | Quadrivalent HPV vaccine (Gardasil) | Placebo | 3 doses at months 0, 2, and 6 | Multinational/22 | 6/36 |
| Palefsky 2011(2) ITT (NCT00090285) Intention-to-Treat Population | Young HIV-negative men who have sex with men from the general population | Quadrivalent HPV vaccine (Gardasil) | Placebo | 3 doses at months 0, 2, and 6 | Multinational/22 | 6/36 |
| Qiao 2020 (NCT01735006) | Healthy adult women from the general population | Bivalent HPV vaccine | Placebo | 3 doses at months 0, 1, and 6 | China/30 | 6/42 |
| Roteli-Martins 2012 (HPV-023, NCT00518336) | Young women from the general population | Bivalent HPV AS04-adjuvanted vaccine (Cervarix) | Placebo | 3 doses at months 0, 1, and 6 | Brazil/26.5 | 6/100.8 |
| Roteli-martins 2012 (4y) (HPV-023, NCT00518336)  y: year | Young women from the general population | Bivalent HPV AS04-adjuvanted vaccine (Cervarix) | Placebo | 3 doses at months 0, 1, and 6 | Brazil/26.5 | 6/100.8 |
| Roteli-martins 2012 (6.4y) (HPV-023, NCT00518336) | Young women from the general population | Bivalent HPV AS04-adjuvanted vaccine (Cervarix) | Placebo | 3 doses at months 0, 1, and 6 | Brazil/26.5 | 6/100.8 |
| Roteli-martins 2012 (8.4y) (HPV-023, NCT00518336) | Young women from the general population | Bivalent HPV AS04-adjuvanted vaccine (Cervarix) | Placebo | 3 doses at months 0, 1, and 6 | Brazil/26.5 | 6/100.8 |
| Sheth 2024 (NCT02864147) | Adult women with high-grade cervical intraepithelial neoplasia | 9-Valent HPV vaccine | Placebo | 2 doses at months 0, 2 | USA/28 | 6/6 |
| Sheth 2024 (E) (NCT02864147)  E: end treatment | Adult women with high-grade cervical intraepithelial neoplasia | 9-Valent HPV vaccine | Placebo | 2 doses at months 0, 2 | USA/28 | 4/6 |
| Sheth 2024 (F) (NCT02864147)  F: follow-up visit | Adult women with high-grade cervical intraepithelial neoplasia | 9-Valent HPV vaccine | Placebo | 2 doses at months 0, 2 | USA/28 | 4/6 |
| Sheth 2024 (IM) (NCT02864147)  IM: imiquimod | Adult women with high-grade cervical intraepithelial neoplasia | 9-Valent HPV vaccine | Placebo | 2 doses at months 0, 2 | USA/28 | 4/6 |
| Sheth 2024 (M) (NCT02864147)  M: mid- treatment | Adult women with high-grade cervical intraepithelial neoplasia | 9-Valent HPV vaccine | Placebo | 2 doses at months 0, 2 | USA/28 | 4/6 |
| Sheth 2024 (SR) (NCT02864147)  SR: surveillance | Adult women with high-grade cervical intraepithelial neoplasia | 9-Valent HPV vaccine | Placebo | 2 doses at months 0, 2 | USA/28 | 4/6 |
| Shi 2023 (NCT02740790) | Healthy females from the general population | Bivalent HPV vaccine | Placebo | 3 doses at months 0, 2, and 6 | China/17.3 | 6/48 |
| Shi 2023 (12m) (NCT02740790) | Healthy females from the general population | Bivalent HPV vaccine | Placebo | 3 doses at months 0, 2, and 6 | China/17.3 | 6/48 |
| Shi 2023 (7m) (NCT02740790) | Healthy females from the general population | Bivalent HPV vaccine | Placebo | 3 doses at months 0, 2, and 6 | China/17.3 | 6/48 |
| Skinner, 2014 (NCT00294047) | Adult women older than 25 years from the general population | Bivalent HPV AS04-adjuvanted vaccine (Cervarix) | Aluminum hydroxide placebo | 3 doses at months 0, 1, and 6 | Multinational/37 | 6/40 |
| Sow 2012 (NCT00481767) | HIV-seronegative girls and young women | Bivalent HPV AS04-adjuvanted vaccine (Cervarix) | Placebo (Aluminum hydroxide Al(OH)₃) | 3 doses at months 0, 1, and 6 | Senegal, Tanzania/16.9 | 6/12 |
| Sow 2012 (SN) (NCT00481767)  SN: seronegative | HIV-seronegative girls and young women | Bivalent HPV AS04-adjuvanted vaccine (Cervarix) | Placebo (Aluminum hydroxide Al(OH)₃) | 3 doses at months 0, 1, and 6 | Senegal, Tanzania/16.9 | 6/12 |
| Sow 2012 (SP) (NCT00481767)  SP: seropositive | HIV-seronegative girls and young women | Bivalent HPV AS04-adjuvanted vaccine (Cervarix) | Placebo (Aluminum hydroxide Al(OH)₃) | 3 doses at months 0, 1, and 6 | Senegal, Tanzania/16.9 | 6/12 |
| Szarewski 2011 (1) (NCT00122681), HPV-16/18 DNA negative regardless of serological status | Young women (15–25 years) from the general population | Bivalent HPV AS04-adjuvanted vaccine (Cervarix) | Hepatitis A vaccine (Havrix) | 3 doses at months 0, 1, and 6 | Multinational/NR | 6/48 |
| Szarewski 2011 (2) (NCT00122681), HPV-16/18 DNA negative and seronegative | Young women (15–25 years) from the general population | Bivalent HPV AS04-adjuvanted vaccine (Cervarix) | Hepatitis A vaccine (Havrix) | 3 doses at months 0, 1, and 6 | Multinational/NR | 6/48 |
| Szarewski 2011 (3) (NCT00122681), HPV-16/18 DNA negative and seropositive | Young women (15–25 years) from the general population | Bivalent HPV AS04-adjuvanted vaccine (Cervarix) | Hepatitis A vaccine (Havrix) | 3 doses at months 0, 1, and 6 | Multinational/NR | 6/48 |
| Szarewski 2011-1 (12m) (NCT00122681) | Young women (15–25 years) from the general population | Bivalent HPV AS04-adjuvanted vaccine (Cervarix) | Hepatitis A vaccine (Havrix) | 3 doses at months 0, 1, and 6 | Multinational/NR | 6/48 |
| Szarewski 2011-1 (6m) (NCT00122681) | Young women (15–25 years) from the general population | Bivalent HPV AS04-adjuvanted vaccine (Cervarix) | Hepatitis A vaccine (Havrix) | 3 doses at months 0, 1, and 6 | Multinational/NR | 6/48 |
| Szarewski 2011-2(12m) (NCT00122681) | Young women (15–25 years) from the general population | Bivalent HPV AS04-adjuvanted vaccine (Cervarix) | Hepatitis A vaccine (Havrix) | 3 doses at months 0, 1, and 6 | Multinational/NR | 6/48 |
| Szarewski 2011-2(6m) (NCT00122681) | Young women (15–25 years) from the general population | Bivalent HPV AS04-adjuvanted vaccine (Cervarix) | Hepatitis A vaccine (Havrix) | 3 doses at months 0, 1, and 6 | Multinational/NR | 6/48 |
| Szarewski 2011-3(12m) (NCT00122681) | Young women (15–25 years) from the general population | Bivalent HPV AS04-adjuvanted vaccine (Cervarix) | Hepatitis A vaccine (Havrix) | 3 doses at months 0, 1, and 6 | Multinational/NR | 6/48 |
| Szarewski 2011-3(6m) (NCT00122681) | Young women (15–25 years) from the general population | Bivalent HPV AS04-adjuvanted vaccine (Cervaix) | Hepatitis A vaccine (Havrix) | 3 doses at months 0, 1, and 6 | Multinational/NR | 6/48 |
| Wheeler 2012 (NCT00122681) | Young women (15–25 years) from the general population | Bivalent HPV AS04-adjuvanted vaccine (Cervarix) | Hepatitis A vaccine (Havrix) | 3 doses at months 0, 1, and 6 | Multinational/NR | 6/48 |
| Wheeler 2022-1(48m) (NCT00294047) | Young women (15–25 years) from the general population | Bivalent HPV AS04-adjuvanted vaccine (Cervarix) | Aluminum hydroxide placebo | 3 doses at months 0, 1, and 6 | Multinational/NR | 6/84 |
| Wheeler 2022-1(84m) (NCT00294047) | Young women (15–25 years) from the general population | Bivalent HPV AS04-adjuvanted vaccine (Cervarix) | Aluminum hydroxide placebo | 3 doses at months 0, 1, and 6 | Multinational/NR | 6/84 |
| Wheeler 2022-2(48m) (NCT00294047) | Young women (15–25 years) from the general population | Bivalent HPV AS04-adjuvanted vaccine (Cervarix) | Aluminum hydroxide placebo | 3 doses at months 0, 1, and 6 | Multinational/NR | 6/84 |
| Wheeler 2022-2(84m) (NCT00294047) | Young women (15–25 years) from the general population | Bivalent HPV AS04-adjuvanted vaccine (Cervarix) | Aluminum hydroxide placebo | 3 doses at months 0, 1, and 6 | Multinational/NR | 6/84 |
| Zhao 2022 (NCT01735006) | Adult women from the general population | Bivalent HPV vaccine | Hepatitis E vaccine (Hecolin) | 3 doses at months 0, 1, and 6 | China/30 | 6/66 |
| Zhao 2022 (1) (NCT01735006) PPS-PI=per-protocol set | Adult women from the general population | Bivalent HPV vaccine | Hepatitis E vaccine (Hecolin) | 3 doses at months 0, 1, and 6 | China/30 | 6/66 |
| Zhao 2022 (2) (NCT01735006) mITT-E=modified intention-to-treat analysis | Adult women from the general population | Bivalent HPV vaccine | Hepatitis E vaccine (Hecolin) | 3 doses at months 0, 1, and 6 | China/30 | 6/66 |
| Zhao 2022 (3) (NCT01735006) ITT-E=intention-to-treat analysis | Adult women from the general population | Bivalent HPV vaccine | Hepatitis E vaccine (Hecolin) | 3 doses at months 0, 1, and 6 | China/30 | 6/66 |
| Zhao-c 2022 (NCT01735006) | Adult women from the general population | Quadrivalent HPV vaccine (Gardasil) | Placebo | 3 doses at months 0, 2, and 6 | China/30 | 6/94 |
| Zhu 2014 (NCT00779766) | Young healthy Chinese women from the general population | Bivalent HPV AS04-adjuvanted vaccine (Cervarix) | Aluminum hydroxide (Al(OH)3) placebo | 3 doses at months 0, 1, and 6 | China/23 | 6/15 |
| Zhu 2014 (12m) (NCT00779766) | Young healthy Chinese women from the general population | Bivalent HPV AS04-adjuvanted vaccine (Cervarix) | Aluminum hydroxide (Al(OH)3) placebo | 3 doses at months 0, 1, and 6 | China/23 | 6/15 |
| Zhu 2014 (6m) (NCT00779766) | Young healthy Chinese women from the general population | Bivalent HPV AS04-adjuvanted vaccine (Cervarix) | Aluminum hydroxide (Al(OH)3) placebo | 3 doses at months 0, 1, and 6 | China/23 | 6/15 |
| Zhu 2016 (NCT00779766) | Young healthy Chinese women from the general population | Bivalent AS04-HPV vaccine | Aluminum hydroxide placebo | 3 doses at months 0, 1, and 6 | China/23 | 6/57 |
| Zhu 2016 (1) (NCT00779766) ATP-E, according-to-protocol cohort for efficacy | Young healthy Chinese women from the general population | Bivalent AS04-HPV vaccine | Aluminum hydroxide placebo | 3 doses at months 0, 1, and 6 | China/23 | 6/57 |
| Zhu 2016 (2) (NCT00779766) TVC-E, total vaccinated cohort for efficacy | Young healthy Chinese women from the general population | Bivalent AS04-HPV vaccine | Aluminum hydroxide placebo | 3 doses at months 0, 1, and 6 | China/23 | 6/57 |
| Zhu 2016-1 (12m) (NCT00779766) | Young healthy Chinese women from the general population | Bivalent AS04-HPV vaccine | Aluminum hydroxide placebo | 3 doses at months 0, 1, and 6 | China/23 | 6/57 |
| Zhu 2016-1 (6m) (NCT00779766) | Young healthy Chinese women from the general population | Bivalent AS04-HPV vaccine | Aluminum hydroxide placebo | 3 doses at months 0, 1, and 6 | China/23 | 6/57 |
| Zhu 2016-2 (12m) (NCT00779766) | Young healthy Chinese women from the general population | Bivalent AS04-HPV vaccine | Aluminum hydroxide placebo | 3 doses at months 0, 1, and 6 | China/23 | 6/57 |
| Zhu 2016-2 (6m) (NCT00779766) | Young healthy Chinese women from the general population | Bivalent AS04-HPV vaccine | Aluminum hydroxide placebo | 3 doses at months 0, 1, and 6 | China/23 | 6/57 |
| Zhu 2019 (NCT00779766) | Young healthy Chinese women from the general population | Bivalent AS04-HPV vaccine | Aluminum hydroxide placebo | 3 doses at months 0, 1, and 6 | China/23 | 6/72 |
| Zhu 2019 (1) (NCT00779766) ATP‐E | Young healthy Chinese women from the general population | Bivalent AS04-HPV vaccine | Aluminum hydroxide placebo | 3 doses at months 0, 1, and 6 | China/23 | 6/72 |
| Zhu 2019 (2) (NCT00779766) TVCE | Young healthy Chinese women from the general population | Bivalent AS04-HPV vaccine | Aluminum hydroxide placebo | 3 doses at months 0, 1, and 6 | China/23 | 6/72 |
| Zhu 2019-1 (12m) (NCT00779766) | Young healthy Chinese women from the general population | Bivalent AS04-HPV vaccine | Aluminum hydroxide placebo | 3 doses at months 0, 1, and 6 | China/23 | 6/72 |
| Zhu 2019-1 (6m) (NCT00779766) | Young healthy Chinese women from the general population | Bivalent AS04-HPV vaccine | Aluminum hydroxide placebo | 3 doses at months 0, 1, and 6 | China/23 | 6/72 |
| Zhu 2019-2 (12m) (NCT00779766) | Young healthy Chinese women from the general population | Bivalent AS04-HPV vaccine | Aluminum hydroxide placebo | 3 doses at months 0, 1, and 6 | China/23 | 6/72 |
| Zhu 2019-2 (6m) (NCT00779766) | Young healthy Chinese women from the general population | Bivalent AS04-HPV vaccine | Aluminum hydroxide placebo | 3 doses at months 0, 1, and 6 | China/23 | 6/72 |

^†^HPV: Human Papillomavirus, m: month.

**Table S3.** Egger's test results for assessing publication bias.

| Outcome | No. of studies | Egger's Intercept | p-value |
| --- | --- | --- | --- |
| ASC-US | 12 | -1.500354 | **0.004^*^** |
| CIN I | 21 | -0.3454539 | **0.04^*^** |
| CIN II | 24 | -0.2471246 | 0.057 |
| GMT16 | 17 | -161.2102 | 0.297 |
| GMT18 | 20 | 442.6158 | **0.005^*^** |
| Grade 3 AE | 10 | 2.185477 | 0.565 |
| HSIL | 13 | 0.2457119 | 0.573 |
| Incident infections | 24 | -1.427996 | **0.001^*^** |
| Injection-site AE | 12 | 2.620588 | 0.164 |
| LSIL | 10 | -0.6565842 | 0.178 |
| Persistent infections | 50 | -0.6644322 | **0.008^*^** |
| Serious AE | 20 | 0.4738353 | 0.291 |
| Systemic AE | 11 | -0.6190298 | 0.126 |

ASC-US: atypical squamous cells of undetermined significance; CIN: cervical intraepithelial neoplasia grade; HSIL: high-grade squamous intraepithelial lesion; LSIL: low-grade squamous intraepithelial lesion; GMT16: geometric mean titer of anti HPV16 antibody; GMT18: geometric mean titer of anti HPV18 antibody, AE: adverse event. ^*^ p -value **<** 0.05

**Table 4.** Trim and fill analysis results.

| Outcome | Model | Observed Effect Size (95% CI) | Imputed Studies | Adjusted Effect Size (95% CI) |
| --- | --- | --- | --- | --- |
| ASC-US | Random-effects | 0.703 (0.533–0.872) | 0 | 0.703 (0.533–0.872) |
| CIN I | Random-effects | 0.357 (0.225–0.490) | 0 | 0.357 (0.225–0.490) |
| GMT 18 | Random-effects | 2.780 (1.969–3.590) | 0 | 2.780 (1.969–3.590) |
| Incident infections | Random-effects | 0.326 (0.186–0.467) | 0 | 0.326 (0.186–0.467) |
| Persistent infections | Random-effects | 0.371 (0.272–0.470) | 0 | 0.371 (0.272–0.470) |

**Table 5.** Meta-regression analysis of main outcomes.

| Variable | Coefficient | Standard Error | p-value | t-value | 95% Confidence Interval | Tau2 |
| --- | --- | --- | --- | --- | --- | --- |
| ASC-US | | | | | | |
| Age | 0.0748 | 0.1929 | 0.706 | 0.39 | [-0.3552, 0.5048] | 0.141 |
| HIV Status | 0.5069 | 1.0881 | 0.651 | 0.47 | [-1.9174, 2.9313] | 0.1167 |
| Vaccine Type | -0.1635 | 0.7001 | 0.820 | -0.23 | [-1.7235, 1.3965] | 0.1232 |
| CIN I | | | | | | |
| Age | 0.1963 | 0.1178 | 0.112 | 1.67 | [-0.0503, 0.4429] | 0 |
| HIV Status | -0.5122 | 0.3330 | 0.141 | -1.54 | [-1.2093, 0.1848] | 0 |
| Dose | -0.5542 | 0.3412 | 0.121 | -1.62 | [-1.2684, 0.1599] | 0 |
| Vaccine Type | 0.2699 | 0.1696 | 0.128 | 1.59 | [-0.0851, 0.6250] | 0 |
| CIN II | | | | | | |
| Age | 0.1135 | 0.1240 | 0.370 | 0.92 | [-0.1436, 0.3706] | 0 |
| HIV Status | -0.6922 | 0.8025 | 0.398 | -0.86 | [-2.3570, 0.9726] | 0 |
| Dose | -1.0787 | 0.9440 | 0.265 | -1.14 | [-3.0364, 0.8790] | 0 |
| Vaccine Type | 1.0787 | 0.9440 | 0.265 | 1.14 | [-0.8789, 3.0364] | 0 |
| GMT16 | | | | | | |
| Age | 0.9481 | 0.9356 | 0.325 | 1.01 | [-1.0258, 2.9219] | 6.813 |
| HIV Status | 0.8027 | 0.6576 | 0.239 | 1.22 | [-0.5847, 2.1900] | 6.657 |
| Dose | 1.1714 | 1.4922 | 0.443 | 0.79 | [-1.9769, 4.3197] | 6.983 |
| Vaccine Type | 0.9977 | 1.6792 | 0.560 | 0.59 | [-2.5451, 4.5405] | 7.089 |
| Gender | 3.1621 | 2.6561 | 0.250 | 1.19 | [-2.4417, 8.7659] | 6.673 |
| GMT18 | | | | | | |
| Age | -0.0553 | 0.7963 | 0.945 | -0.07 | [-1.7281, 1.6176] | 6.417 |
| HIV Status | 1.328 | 0.7746 | 0.104 | 1.71 | [-0.2994, 2.9554] | 5.495 |
| Dose | 1.4403 | 1.3817 | 0.311 | 1.04 | [-1.4625, 4.3430] | 6.044 |
| Vaccine Type | 1.3300 | 1.3880 | 0.351 | 0.96 | [-1.5863, 4.2458] | 6.1 |
| Gender | 1.1940 | 1.1555 | 0.315 | 1.03 | [-1.2336, 3.6215] | 6.049 |
| Grade 3 AE | | | | | | |
| Age | 0.5523 | 0.6803 | 0.440 | 0.81 | [-1.0165, 2.1211] | 1.44 |
| HIV Status | -0.3776 | 0.4636 | 0.439 | -0.81 | [-1.4467, 0.6915] | 1.383 |
| Dose | 1.5066 | 1.5230 | 0.352 | 0.99 | [-2.0054, 5.0186] | 1.492 |
| Vaccine Type | -0.5065 | 0.5060 | 0.346 | -1.00 | [-1.6732, 0.6602] | 1.536 |
| Gender | 0.0162 | 0.7182 | 0.983 | 0.02 | [-1.6400, 1.6725] | 1.682 |
| Incident Infections | | | | | | |
| Age | -0.2188 | 0.0991 | 0.038 | -2.21 | [-0.4245, -0.0132] | 0.01469 |
| HIV Status | 0.2791 | 0.6329 | 0.664 | 0.44 | [-1.0336, 1.5917] | 0.02899 |
| Dose | -0.1081 | 0.6064 | 0.860 | -0.18 | [-1.3656, 1.1494] | 0.02918 |
| Vaccine Type | -0.1734 | 0.2974 | 0.566 | -0.58 | [-0.7902, 0.4434] | 0.02935 |
| Gender | -0.1734 | 0.2974 | 0.566 | -0.58 | [-0.7902, 0.4434] | 0.02935 |
| Injection-Site AE | | | | | | |
| Age | -0.1037 | 0.1777 | 0.572 | -0.58 | [-0.4997, 0.2923] | 0.1683 |
| HIV Status | 0.1268 | 0.3471 | 0.722 | 0.37 | [-0.6466, 0.9002] | 0.1804 |
| Vaccine Type | 0.4308 | 0.1190 | 0.005 | 3.62 | [0.1657, 0.6960] | 0.06088 |
| LSIL | | | | | | |
| Age | 0.1624 | 0.0872 | 0.099 | 1.86 | [-0.0386, 0.3634] | 0.006641 |
| HIV Status | 0.7549 | 1.0467 | 0.491 | 0.72 | [-1.6589, 3.1688] | 0.01716 |
| Vaccine Type | 0.3498 | 0.1767 | 0.083 | 1.98 | [-0.0576, 0.7572] | 0.006928 |
| Gender | 0.3369 | 0.1750 | 0.090 | 1.92 | [-0.0667, 0.7405] | 0.006738 |
| Persistent Infections | | | | | | |
| Age | 0.0739 | 0.0859 | 0.394 | 0.86 | [-0.0989, 0.2467] | 0.04675 |
| HIV Status | 0.3186 | 1.0359 | 0.760 | 0.31 | [-1.7642, 2.4014] | 0.04763 |
| Vaccine Type | -0.0972 | 0.2189 | 0.659 | -0.44 | [-0.5372, 0.3429] | 0.04864 |
| Dose | 0.0614 | 0.3174 | 0.847 | 0.19 | [-0.5768, 0.6995] | 0.04945 |
| Gender | -0.0846 | 0.1343 | 0.532 | -0.63 | [-0.3546, 0.1854] | 0.04809 |
| Systemic AE | | | | | | |
| Age | -0.005911 | 0.0450673 | 0.899 | -0.13 | [-0.1078603, 0.0960382] | 0.000 |
| HIV Status | 0.0116961 | 0.0640965 | 0.859 | 0.18 | [-0.1333003, 0.1566924] | 0.000 |
| Vaccine Type | 0.0495741 | 0.0497507 | 0.345 | 1.00 | [-0.0629699, 0.1621181] | 0.000 |
| Dose | -0.0267798 | 0.0774097 | 0.737 | -0.35 | [-0.2018927, 0.1483331] | 0.000 |
| Gender | 0.0212617 | 0.0421931 | 0.626 | 0.50 | [-0.0741857, 0.1167092] | 0.000 |

**Table S6.** GRADE assessment of certainty and importance of outcomes.

| Outcome | No of studies | Risk of Bias  (RoB 2 tool) | Inconsistency (I2) | Indirectness | Imprecision | Publication Bias  (Eggers) | No of patients | | Certainty | Importance |
| --- | --- | --- | --- | --- | --- | --- | --- | --- | --- | --- |
|  |  |  |  |  |  |  | Exp | Ctrl |  |  |
| CIN I | 21 | Not serious | Serious ^a^ | Not serious | Not serious | Serious ^b^ | 69332 | 68746 | ⊕⊕◯◯ (Low) | Critical |
| CIN II | 24 | Not serious | Serious ^a^ | Not serious | Not serious | Not serious | 84762 | 83875 | ⊕⊕⊕◯ (Moderate) | Critical |
| CIN III | 5 | Not serious | Serious ^a^ | Not serious | Not serious | Undetected ^c^ | 28314 | 28316 | ⊕⊕⊕◯ (Moderate) | Important |
| Persistent Infections | 51 | Not serious | Serious ^a^ | Not serious | Not serious | Serious ^b^ | 129853 | 129799 | ⊕⊕◯◯ (Low) | Important |
| Incident Infections | 24 | Not serious | Serious ^a^ | Not serious | Not serious | Serious ^b^ | 41288 | 43375 | ⊕⊕◯◯ (Low) | Important |
| ASC-US | 10 | Not serious | Serious ^a^ | Not serious | Not serious | Serious ^b^ | 20018 | 19885 | ⊕⊕◯◯ (Low) | Critical |
| HSIL | 14 | Not serious | Not serious | Not serious | Not serious | Not serious | 29192 | 29077 | ⊕⊕⊕⊕ (High) | Important |
| LSIL | 8 | Not serious | Serious ^a^ | Not serious | Not serious | Undetected ^c^ | 27176 | 27035 | ⊕⊕⊕◯ (Moderate) | Important |
| AIN | 5 | Not serious | Not serious | Not serious | Not serious | Undetected ^c^ | 781 | 800 | ⊕⊕⊕⊕ (High) | Critical |
| AIS | 3 | Not serious | Not serious | Not serious | Not serious | Undetected ^c^ | 17849 | 1783 | ⊕⊕⊕⊕ (High) | Important |
| EGL | 7 | Not serious | Serious ^a^ | Not serious | Not serious | Undetected ^c^ | 3500 | 3482 | ⊕⊕⊕◯ (Moderate) | Important |
| TCD4+ | 4 | Not serious | Serious ^a^ | Not serious | Not serious | Undetected ^c^ | 154 | 149 | ⊕⊕⊕◯ (Moderate) | Important |
| TCD8+ | 3 | Not serious | Serious ^a^ | Not serious | Serious ^d^ | Undetected ^c^ | 93 | 90 | ⊕⊕◯◯ (Low) | Important |
| GMT6 | 4 | Not serious | Serious ^a^ | Not serious | Not serious | Undetected ^c^ | NA | NA | ⊕⊕⊕◯ (Moderate) | Important |
| GMT11 | 4 | Not serious | Serious ^a^ | Not serious | Not serious | Undetected ^c^ | NA | NA | ⊕⊕⊕◯ (Moderate) | Critical |
| GMT16 | 19 | Not serious | Serious ^a^ | Not serious | Not serious | Not serious | NA | NA | ⊕⊕⊕◯ (Moderate) | Critical |
| GMT18 | 20 | Not serious | Serious ^a^ | Not serious | Not serious | Serious ^b^ | NA | NA | ⊕⊕◯◯ (Low) | Critical |
| Grade 3 AE | 15 | Not serious | Serious ^a^ | Not serious | Not serious | Not serious | 12103 | 11777 | ⊕⊕◯◯ (Low) | Critical |
| Serious AE | 27 | Not serious | Not serious | Not serious | Not serious | Not serious | 26529 | 26354 | ⊕⊕⊕⊕ (High) | Important |
| Injection-Site AE | 12 | Not serious | Serious ^a^ | Not serious | Not serious | Not serious | 12574 | 12218 | ⊕⊕⊕◯ (Moderate) | Important |
| Systemic AE | 11 | Not serious | Serious ^a^ | Not serious | Not serious | Not serious | 9693 | 9347 | ⊕⊕⊕◯ (Moderate) | Important |
| Elective termination | 8 | Not serious | Not serious | Not serious | Not serious | Undetected ^c^ | 16428 | 16182 | ⊕⊕⊕⊕ (High) | Critical |
| Live Birth | 5 | Not serious | Not serious | Not serious | Not serious | Undetected ^c^ | 7599 | 7373 | ⊕⊕⊕⊕ (High) | Critical |
| Spontaneous Abortion | 8 | Not serious | Not serious | Not serious | Not serious | Undetected ^c^ | 16428 | 16182 | ⊕⊕⊕⊕ (High) | Critical |
| Ectopic Pregnancy | 5 | Not serious | Not serious | Not serious | Not serious | Undetected ^c^ | 10429 | 10192 | ⊕⊕⊕⊕ (High) | Critical |
| Congenital Anomalies | 6 | Not serious | Not serious | Not serious | Not serious | Undetected ^c^ | 13262 | 13117 | ⊕⊕⊕⊕ (High) | Critical |
| Stillbirth | 7 | Not serious | Not serious | Not serious | Not serious | Undetected ^c^ | 14171 | 13925 | ⊕⊕⊕⊕ (High) | Critical |
| PTB | 2 | Not serious | Not serious | Not serious | Not serious | Undetected ^c^ | 962 | 2384 | ⊕⊕⊕⊕ (High) | Critical |

^a^ Serious inconsistency due to high heterogeneity (I² > 50%).

^b^ Serious concern of publication bias due to asymmetry in the funnel plot and significant Egger’s test results.

^c^ Fewer than 10 studies, so publication bias could not be reliably assessed.
^d^ Due to a wide confidence interval crossing the null effect and a small sample size.

**Table S7.** Summary of main clinical, immunogenicity, and safety outcomes of prophylactic HPV vaccination.

| Outcome | Direction of Effect | Effect Size (95% CI) | Certainty of Evidence (GRADE) | Clinical Importance |
| --- | --- | --- | --- | --- |
| CIN I | ↓ Risk | RR = 0.15 (0.09–0.24) | Low | Critical |
| CIN II | ↓ Risk | RR = 0.20 (0.13–0.30) | Moderate | Critical |
| CIN III | ↓ Risk | RR = 0.48 (0.23–0.98) | Moderate | Important |
| Persistent HPV16/18 infection | ↓ Risk | RR = 0.16 (0.12–0.21) | Low | Important |
| Incident HPV16/18 infection | ↓ Risk | RR = 0.25 (0.19–0.34) | Low | Important |
| ASC-US | ↓ Risk | RR = 0.31 (0.22–0.42) | Low | Critical |
| HSIL | ↓ Risk | RR = 0.82 (0.68–1.00) | High | Important |
| LSIL | ↓ Risk | RR = 0.63 (0.49–0.81) | Moderate | Important |
| AIN | ↓ Risk | RR = 0.64 (0.51–0.80) | High | Critical |
| AIS | ↓ Risk | RR = 0.32 (0.11–0.98) | High | Important |
| EGL | No significant effect | RR = 0.66 (0.29–1.49) | Moderate | Important |
| Tissue CD4⁺ T cells | ↓ Level | SMD = −1.27 (−2.24 to −0.31) | Moderate | Important |
| Tissue CD8⁺ T cells | No significant effect | SMD = −1.12 (−2.75 to 0.51) | Low | Important |
| GMT HPV6 | ↑ Antibody response | Ratio = 3.48 (2.59–4.37) | Moderate | Important |
| GMT HPV11 | ↑ Antibody response | Ratio = 3.37 (1.79–4.96) | Moderate | Critical |
| GMT HPV16 | ↑ Antibody response | Ratio = 3.09 (2.16–4.03) | Moderate | Critical |
| GMT HPV18 | ↑ Antibody response | Ratio = 3.10 (2.28–3.92) | Low | Critical |
| Grade 3 adverse events | No significant difference | RR = 1.42 (0.76–2.64) | Low | Critical |
| Serious adverse events | ↓ Risk | RR = 0.90 (0.82–0.99) | High | Important |
| Injection-site adverse events | ↑ Risk | RR = 1.26 (1.07–1.48) | Moderate | Important |
| Systemic adverse events | No significant difference | RR = 0.99 (0.92–1.06) | Moderate | Important |
| Elective termination | No significant difference | RR = 0.99 (0.93–1.05) | High | Critical |
| Live birth | No significant difference | RR = 1.01 (0.95–1.06) | High | Critical |
| Spontaneous abortion | No significant difference | RR = 1.04 (0.92–1.18) | High | Critical |
| Ectopic pregnancy | No significant difference | RR = 0.77 (0.44–1.36) | High | Critical |
| Congenital anomalies | No significant difference | RR = 1.05 (0.66–1.68) | High | Critical |
| Stillbirth | No significant difference | RR = 0.67 (0.35–1.31) | High | Critical |
| Preterm birth (PTB) | No significant difference | RR = 0.78 (0.51–1.20) | High | Critical |
