# Supplementary material for: Comprehensive evaluation of prophylactic HPV vaccines: a systematic review and meta-analysis of efficacy, safety, and immunogenicity in males and females
Source: Front Immunol. 2026 Jan 13;16:1747082. doi: 10.3389/fimmu.2025.1747082 (PMC12835409; doi:10.3389/fimmu.2025.1747082)
Supplement: Supplementary file 2 [file DataSheet1.docx]

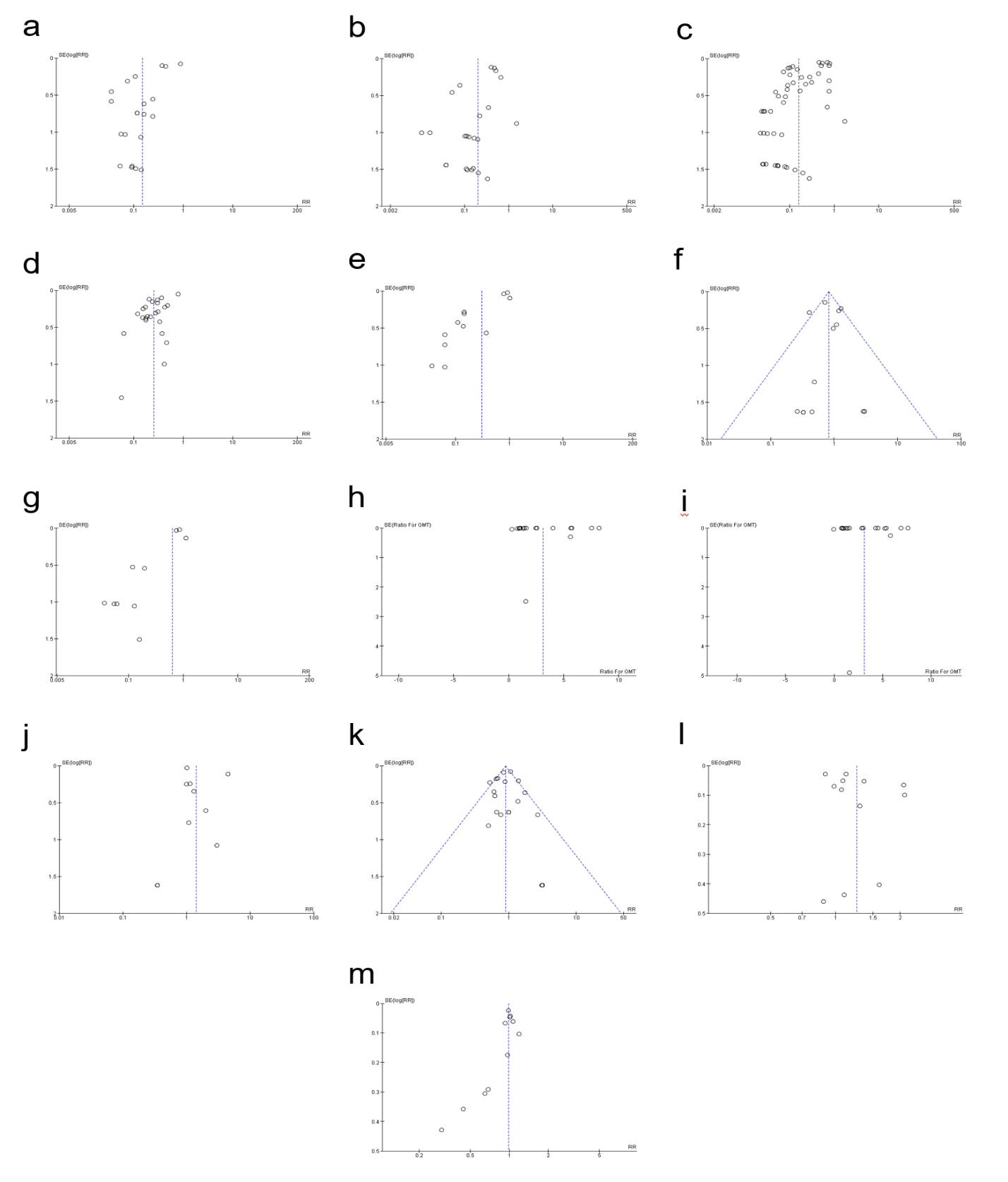


**Figure S1.** Funnel plot of the a) CIN I, b) CIN II, c) persistent infections d) incident infections e) ASC-US, f) HSIL g) LSIL, h) GMT16, i) GMT18 j) grade 3 adverse events k) serious adverse events, l) injection-site adverse events and m) systemic adverse events. ASC-US: atypical squamous cells of undetermined significance; CIN: cervical intraepithelial neoplasia grade; HSIL: high-grade squamous intraepithelial lesion; LSIL: low-grade squamous intraepithelial lesion; GMT16: geometric mean titer of anti HPV16 antibody; GMT18: geometric mean titer of anti HPV18 antibody.

**Figure S2.** Subgroup analysis of CIN I based on a) age, b) dose schedule, c) HIV status and d) vaccine type. HIVN: HIV negative. NR: not reported.

**Figure S3.** Subgroup analysis of CIN II based on a) age, b) dose schedule, c) HIV status and d) vaccine type. HIVN: HIV negative. NR: not reported.

**Figure S4.** Subgroup analysis of persistent infections based on a) age, b) dose schedule, c) HIV status d) vaccine type and e) gender. HIVN: HIV negative. NR: not reported.

**Figure S5.** Subgroup analysis of incident infections based on a) age, b) dose schedule, c) HIV status, d) vaccine type and e) gender. HIVN: HIV negative. NR: not reported.

**Figure S6.** Subgroup analysis of ASC-US based on a) age, b) dose schedule, c) HIV status, d) vaccine type and e) gender. HIVN: HIV negative. NR: not reported. ASC-US: atypical squamous cells of undetermined significance.

**Figure S7.** Subgroup analysis of LSIL based on a) age, b) dose schedule, c) HIV status, d) vaccine type and e) gender. HIVN: HIV negative. NR: not reported. LSIL: low-grade squamous intraepithelial lesion.

**Figure S8.** Subgroup analysis of GMT16 based on a) age, b) dose schedule, c) HIV status, d) vaccine type, e) gender and f) assay method. HIVN: HIV negative. NR: not reported. GMT16: geometric mean titer of anti HPV16 antibody.

**Figure S9.** Subgroup analysis of GMT18 based on a) age, b) dose schedule, c) HIV status, d) vaccine type, e) gender and f) assay method. HIVN: HIV negative. NR: not reported. GMT18: geometric mean titer of anti HPV18 antibody.

**Figure S10.** Subgroup analysis of grade 3 adverse events based on a) age, b) dose schedule, c) HIV status, d) vaccine type, e) gender and f) follow-up duration. HIVN: HIV negative. NR: not reported.

**Figure S11.** Subgroup analysis of injection site adverse events based on a) age, b) dose schedule, c) HIV status, d) vaccine type, e) gender and f) follow-up duration. HIVN: HIV negative. NR: not reported.

**Figure S12.** Subgroup analysis of systemic adverse events based on a) age, b) dose schedule, c) HIV status, d) vaccine type and e) gender. HIVN: HIV negative. NR: not reported.

**Figure S13.** Sensitivity analysis of a) CIN I, b) CIN II, c) persistent infections d) incident infections e) ASC-US, f) HSIL

**Figure S14.** Sensitivity analysis of the a) LSIL, b) GMT16 II, c) GMT18 d) grade 3 adverse events e) serious adverse events, f) injection-site adverse events g) systemic adverse events.
